# Using synthetic bacterial enhancers to reveal a looping-based mechanism for quenching-like repression

**DOI:** 10.1038/ncomms10407

**Published:** 2016-02-02

**Authors:** Michal Brunwasser-Meirom, Yaroslav Pollak, Sarah Goldberg, Lior Levy, Orna Atar, Roee Amit

**Affiliations:** 1 Department of Biotechnology and Food Engineering, Technion—Israel Institute of Technology, Haifa 32000, Israel; 2 Russell Berrie Nanotechnology Institute, Technion—Israel Institute of Technology, Haifa 32000, Israel

## Abstract

We explore a model for ‘quenching-like' repression by studying synthetic bacterial enhancers, each characterized by a different binding site architecture. To do so, we take a three-pronged approach: first, we compute the probability that a protein-bound dsDNA molecule will loop. Second, we use hundreds of synthetic enhancers to test the model's predictions in bacteria. Finally, we verify the mechanism bioinformatically in native genomes. Here we show that excluded volume effects generated by DNA-bound proteins can generate substantial quenching. Moreover, the type and extent of the regulatory effect depend strongly on the relative arrangement of the binding sites. The implications of these results are that enhancers should be insensitive to 10–11 bp insertions or deletions (INDELs) and sensitive to 5–6 bp INDELs. We test this prediction on 61 σ^54^-regulated *qrr* genes from the *Vibrio* genus and confirm the tolerance of these enhancers' sequences to the DNA's helical repeat.

## Abstract (web summary)

Some repressors down-regulate gene expression when they bind to a site up to 100 base pairs away from the nearest activator. Here, the authors show that this quenching-like repression can be achieved by the excluded volume effect when a DNA-bound protein alters the propensity of DNA to form a loop.

## Graphical abstract

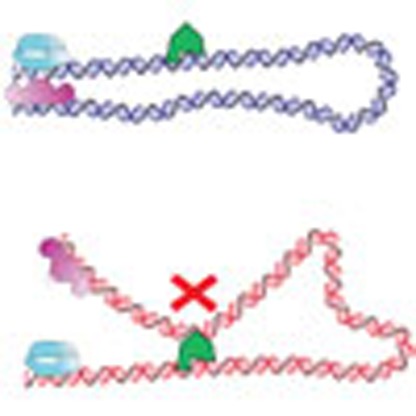

Distal regulation by transcription factors, which are bound *in cis*, yet some distance away from the core promoters, is a regulatory phenomenon ubiquitous in all organisms^1^,^2^,^3^,^4^. Mechanistically, DNA looping has been implicated in distal regulation in eukaryotes^5^,^6^,^7^ and has been shown directly to be involved in σ^54^ promoter expression in bacteria^8^. However, most structural features of distal regulatory regions such as the importance of having several binding sites for a given transcription factor, the genomic distance of these binding sites from the basal promoter and the functional significance of particular arrangements of the binding sites remain poorly understood.

In bacteria, distal regulatory regions called ‘bacterial enhancers' are positioned within 200 bp of a σ^54^ promoter and seem to fall into one of two broad classes. The enhancers in the first class contain activator-binding sites and an additional site for the ubiquitous nucleoid-associated protein integration host factor (IHF)^9^,^10^. The enhancers in the second class do not contain IHF-binding sites, but either harbour additional sites for other transcription factors^11^,^12^,^13^ or exhibit some conserved AT-rich sequence^14^,^15^. In eukaryotes, the diversity of distal regulatory regions is vastly richer. There are several well-known examples of ‘promoter-proximal' distal regulatory regions, which are clusters of transcription factor-binding sites that are located within 100–300 bp away from a core PolII promoter and are thus similar in sequence length and regulatory content to bacterial enhancers. Examples include the c-*fos* promoter in mammalian cells^16^,^17^, the gal1 promoter in *Saccharomyces cerevisiae*^18^ and the *hb* promoter^19^ in *Drosophila melanogaster*. In addition, in most higher eukaryotes, there is another class of distal regulatory elements, which are often called ‘enhancers'. These regions may be located one kbp to several Mbp away from their regulated promoter and contain clusters of transcription factor-binding sites as well^19^. As most of the sequence for distal regulatory regions (independent of their proximity to the core promoter) is believed to be non-coding, the evolutionary pressure on conservation is small, resulting in highly divergent sequences for homologous promoter-proximal regions and enhancers^20^. Consequently, computationally predicting the regulatory output function for an unannotated distal regulatory region in all organisms has proven to be a difficult task^21^,^22^.

To develop a deeper understanding of distal regulatory regions, not only is there a need for additional gene expression data sets but the underlying mechanistic models must be formulated as well. To address this problem, we present here a model-focused variation of the ‘synthetic enhancer' approach (that is, libraries of bacterial enhancers that are engineered via high-capacity double-stranded DNA synthesis techniques)^8^,^23^, to provide one possible mechanism for ‘quenching-like' repression^20^. ‘Quenching' is a form of repression originally observed in fly enhancers, where repressors such as Snail^24^, Kruppel^25^, Knirps^26^ or Giant^27^ downregulate expression not via a competition with an activator for binding, but rather through having its binding sites positioned several 10 to ∼100 bp away from the nearest activator. ‘Quenching-like' repression effects have also been reported for eukaryotic promoter-proximal regulatory regions and in bacterial enhancers as well. Here, repressors are bound in between the activators and the core promoter in repressed complexes. Well-documented examples include the YY1 repressor in the c-*f*os and other promoters in mammalian cells^16^,^17^; the α2 repressor was found to be co-bound with the Gal4 activator in a tightly repressed complex in *S. cerevisiae*^28^, the glnAp2 σ^54^ promoter in *Escherichia coli*^11^ and the Nac σ^54^ promoter in *Klesbiella aerogenes*^13^, and quenching-like effects have also been attributed to inadequate positioning of an IHF-binding site with respect to the activator or σ^54^ promoter sequences in other promoters as well^29^. However, despite the many observations of closely bound ensembles of proteins on distal regulatory elements, which interact in a repressive manner to regulate gene expression, establishing a broadly applicable mechanistic explanation has remained elusive.

## Results

### Simulating quenching repression

**Figure 1:**
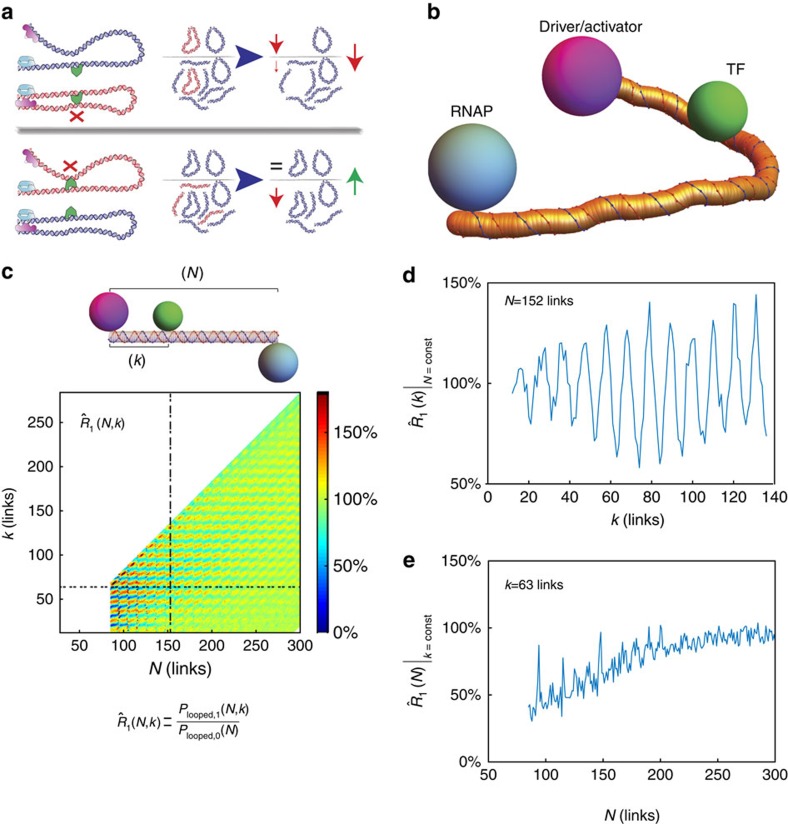
Transcriptional regulation via an excluded volume effect. (**a**) Schematic representation of the looping probability ratio corresponding to the effects of a TF excluded volume on the probability of looping. We focus on two extreme binding configurations: first, the TF is positioned inside the loop ‘in-phase'; thus, its binding site will be separated from the activator's by an integer multiple of the helical repeat (∼*n* × 10.5 bp). Second, the TF is positioned outside of the loop or ‘out-of-phase' from the activator; thus, the binding site separation must be an odd integer multiple of half of the helical repeat (∼(*n*+1/2) × 10.5 bp). In the top schematic (**a**, top), the transcription factor bound ‘in-phase' excludes many of the looped configurations that were available to the polymer not bound by the TF. As a result, the propensity of the bound complex to loop should be reduced as compared with a complex not bound by the TF. This should lead to a repression regulatory effect. Conversely, the ‘out-of-phase' TF is positioned outside of the loop, which mainly results in the exclusion of previously available non-looped configurations. This results in a slight increase in the propensity of the TF bound complex to loop. Consequently, the out-of-phase configuration should yield a small upregulation or activation-like effect (**a**, bottom). Together, the ‘in-phase' and ‘out-of-phase' arrangements of the TF should generate a regulatory response that oscillates between down and upregulation as a function of the position of the TF within the loop. (**b**) Sample conformation of a thick chain with three protrusions generated using the self-avoiding WLC (SAWLC) algorithm. (**c**) Heat-map of the simulated looping probability ratio for a thick chain. Top: schematic with protrusions simulating a minimal enhancer with a single TF-binding site as a function of looping length (*N*) and TF protrusion position (*k*). The size of the TF protrusion was set to be 5.44 nm in diameter. Bottom: schematic showing the definition of the looping probability ratio. (**d**,**e**) One-dimensional cross-sections of the simulated probability ratio heat map at constant *N=*152 bp and *k=*64 bp, respectively.

As quenching is a phenomenon that is associated with proteins that seem to bind DNA several tens of base pairs away from an activator, we hypothesized that the underlying mechanism for repression might be an excluded volume effect, where a bound protein alters the propensity of DNA to form a loop by its mere presence (Fig. 1a). We opted to explore this hypothesis by devising a numerical simulation using the worm-like chain (WLC) model^30^ as a basis (see Supplementary Note 1). To do so, we modified the wormlike chain model to generate chains made of finite volume links (Supplementary Fig. 1a,b). Such ‘thick' chains can be used to probe excluded volume effects, as only configurations where parts of the chain do not cross each other are considered. In addition, thick chains can be ‘deformed' locally by additional volumes or protrusions (Supplementary Fig. 1c,d), and using numerical simulations the effects of these local protrusions on various chain properties can be estimated. Since these protrusions can be likened to proteins bound to DNA, the model's results can be used to estimate the likelihood that a particular protrusion-bound looped configuration will occur. We term this approach the self-avoiding WLC model^31^.

To obtain an initial set of predictions, we generated ensembles containing 10^7^–10^9^ configurations of thick chains with protrusions (see Fig. 1b), up to a chain length of 300 links, with one link corresponding to 1 bp. Each chain in our ensemble was grown one link at a time, avoiding chain–chain collision events via a method termed weighted biased sampling^32^ (see Supplementary Note 2). To model quenching effects, we generated a thick chain architecture containing three protrusions: one at each end of the chain, simulating the activator and holoenzyme complex, respectively, and an additional protrusion simulating a generic transcription factor (TF) positioned somewhere along the chains (Fig. 1b).

Using these ensembles, we computed chain observables. For the case of thick-chain looping, there is considerable freedom in choosing the particular end-to-end separation criterion, which differentiates between looped and non-looped configurations. For the predictions shown in this study, we chose a looping boundary condition (shown in Supplementary Fig. 1e) that mimics the actual geometry of the bacterial σ^54^ interaction with its upstream activator^33^. In our simulations, the TF binding site or protrusion is located some *k* links away from the ‘activator' and *N−k* links away from the promoter (Fig. 1c top). In Fig. 1c middle, we plot the results. The figure is plotted as a heat map representation of the looping probability ratio *R*_1_(*N*,*k*) (Fig. 1c bottom). Each point in the two-dimensional (*N*,*k*) map corresponds to the ratio of the probabilities of looping for an enhancer-mimicking thick chain of length *N* with a TF-like protrusion located *k* links away from the activator, to a thick chain without the TF-like protrusion. The figure shows a two-dimensional oscillatory pattern with alternating patches of upregulation (red, with looping ratio values >100%) and downregulation (blue, looping ratio values <100%) in the probability of looping, with periodicities that are consistent with the 10.5-bp helical periodicity that was preset for our simulation. The value of 100% (yellow–green) corresponds to the case where the looping ratio is one and thus no effect of excluded volume is predicted for those particular binding-site arrangements.

A closer examination of various one-dimensional cross-sections of the heat map shows additional phenomena. For a cross-section at constant *N* (Fig. 1d), the amplitude of the oscillations between the up-regulation maxima and down-regulation minima increases the further the protrusion is positioned away from the chain origin, with a maximal amplitude reached at the position which bisects the chain (*k*≈*N*/2). Furthermore, as the protrusion's location approaches the terminal end of the chain (*k*→*N*), the effect is shifted towards upregulation. Thus, our model predicts that the strongest quenching is achieved not only when the TF is ‘in-phase' (see Fig. 1) with the activator but also at locations inside the loop that are not immediately adjacent to either the activator or promoter, but rather equidistant from both of them. The origin of this effect is rooted in the geometry of the most probable looped configuration, which is shaped like a ‘tear drop'. This shape is characterized by high curvature in the middle of the loop and low curvature in proximity to the chain termini (see Supplementary Fig. 2 and Supplementary Note 4). Finally, the heat map shows that for the cross-sections with constant *k* and varying *N* (Fig. 1e), the looping probability ratio increases initially for smaller looping lengths and saturates at values that are <100% for large *N*.

### Synthetic enhancers with a single binding site

**Figure 2:**
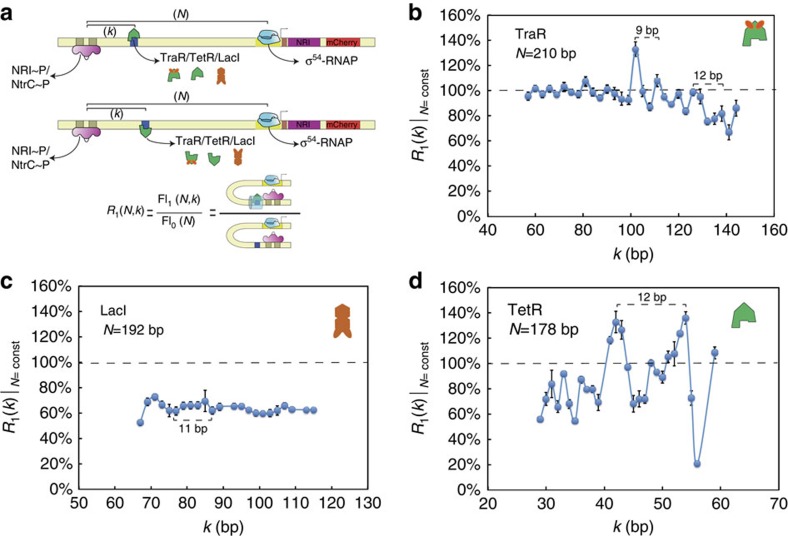
Synthetic enhancers with a single TF-binding site. (**a**) Schematic for the minimal bacterial enhancer system used in our experiments, showing the poised holoenzyme complex at the σ^54^ promoter, NtrC activator and the additional binding site for either TraR, TetR or LacI. The schemas represent the two ‘extreme' configurations. Top: binding sites are positioned ‘out-of-phase' relative to the activator. Bottom: binding sites are positioned ‘in-phase' relative to the activator. The schematic for TraR is drawn with two ovals corresponding to the 3OC8 ligand. (**b**–**d**) Expression level ratio results for synthetic enhancers with a single binding site for TraR (**b**), LacI (**c**) and TetR (**d**) at constant *N*. *k* was varied in 3, 2 and 1 bp steps for TraR, LacI and TetR, respectively. In addition, we show in Supplementary Note 3 that the expression-level ratio observable as defined here is approximately equal to the probability-of-looping ratio given known rates for the NtrC-σ^54^ system, thus allowing us to quantitatively compare experimental results to theoretical predictions. Error bars correspond to the s.d. from multiple measurements.

To test the predictions of the self-avoiding WLC looping model, we constructed libraries of synthetic enhancers in bacteria. Previously, we showed that bacterial enhancers are a unique platform to test distal regulatory models in a synthetic context due to their simplicity of architecture and suitability for high-throughput *in vivo* work^8^. A minimal bacterial enhancer comprises a tandem of activator, or driver, binding sites (for example, NtrC, PspF and so on) located several tens to hundreds of base pairs upstream of a poised σ^54^ promoter (Fig. 2a). These enhancers can be made systematically more complex by adding cassettes of binding sites for other transcription factors in various configurations. We measure our synthetic enhancers' regulatory output via an expression-level assay, where a minimal NRI/NtrC-σ^54^ enhancer^34^ drives the expression of an mCherry reporter, which is encoded downstream from the σ^54^ promoter.

Our first library design consisted of 81 regulatory sequences containing a single binding site for one of three different TFs: LacI^35^, TetR^36^ and TraR^37^. The choice of LacI, TetR and TraR was predicated on their ability to bind DNA dependent on the absence (LacI and TetR) or presence (TraR) of a ligand, whose concentration we controlled externally. In addition, we varied two additional control parameters: the looping length *N*, defined as the distance from the centre of the NtrC/NRI activator-binding site tandem to the centre of the σ^54^ promoter, and the distance *k* separating the centre of the transcription factor-binding site from the middle of the NtrC/NRI-binding site tandem. We limited our enhancers to looping lengths *N*>150 bp but did not restrict the binding site address *k*, allowing its bound TF to be positioned on the same or the opposite DNA face with respect to the activator and the σ^54^, or at some intermediate position.

In Fig. 2b–d, we plot expression-level ratio *R*_1_(*N*,*k*) for constant looping length (*N*) as a function of binding-site position (*k*). *R*_1_(*N*,*k*) is defined as the ratio of expression levels between a protein-bound enhancer and that of an unbound enhancer, for synthetic enhancers containing a single binding site for the three transcription factors: TraR (Fig. 2b), LacI (Fig. 2c) and TetR (Fig. 2d; see Methods and Supplementary Note 3 for a detailed definition). In addition, for all three data sets the position of the binding site (*k*) was varied across the looping region in 1–3 bp intervals. For the TetR and TraR cases, we observe a long-range oscillatory function in the expression-level ratio between quenching and upregulation with a period ∼10–11 bp, which is consistent with the accepted value for the DNA helical repeat (∼10.5–10.9 bp (refs 38, 39, 40, 41)). Here we define quenching as expression-level ratio values that are <100%, as the protein-bound case yields a lower total mCherry reporter level than the unbound case. Conversely, we define upregulation as the case in which the expression-level ratio is >100%. Interestingly, maximal quenching seems to occur at *k-*values that are roughly integer multiples of the DNA helical repeat, whereas binding-site positions that are displaced 5–6 bp away from the minimas resulted in either weaker quenching repression or slight upregulation (>100%) of the expression level of the bound enhancer with respect to the unbound case.

A closer examination of the regulatory response curves shows distinct differences that are strongly dependent on the TF type. For TraR synthetic enhancers (Fig. 2b), the effect of the transcription factor on the probability of looping is small and the total regulatory effect observed varies between weak quenching to slight upregulation (∼75–120%). For LacI synthetic enhancers (Fig. 2c), a barely detectible oscillatory behaviour with ∼11 bp periodicity is observed. Here, the small-amplitude oscillations vary between intermediate (∼50%) to weak quenching (∼70%). Moreover, the amplitude of the oscillations seems to diminish as *k* increases, settling on an intermediate quenching level of ∼60%. Finally, a third distinct regulatory response is observed for TetR in Fig. 2d. Here, regulatory effects persist for the entire segment of the loop tested and both significant quenching and upregulation effects are observed (20–140%). Thus, although the oscillatory quenching/upregulation phenomenon observed clearly for two of the three proteins (TraR and TetR) supports the excluded volume looping-based regulatory mechanism, the differences in the expression-level ratio responses suggest that additional protein-specific aspects need to be added to the model, to better explain the data.

### Additivity in synthetic enhancers

We reasoned that the simplest protein-specific parameter that can affect the regulatory response is the total volume in the loop, as both TetR (25 kDa) and TraR (26 kDa) are significantly smaller than LacI (38 kDa). To try to compensate for the mass and volume difference, we hypothesized that an increase in the number of binding sites for a smaller protein such as TraR should lead to a larger cumulative quenching effect, which will be comparable to the maximal affect achieved by LacI.

**Figure 3:**
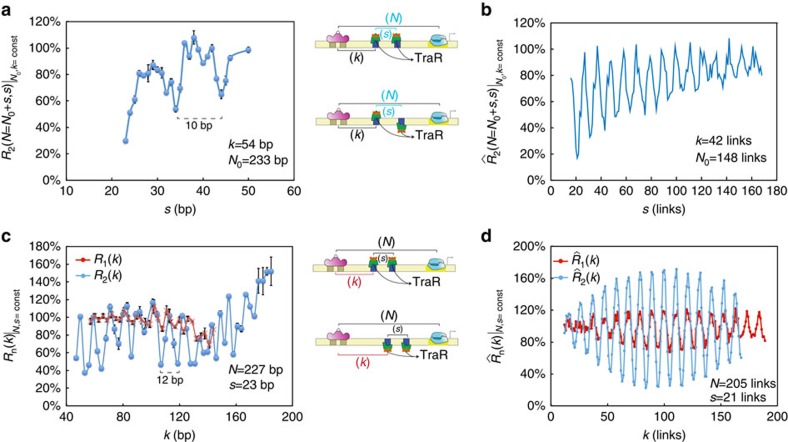
Excluded volume is additive. (**a**) Expression level ratio for synthetic enhancers with a tandem of TraR-binding sites at various inter-TF spacing (*s*) and looping lengths (*N*); see schematic on the right. (**b**) Model predictions for looping probability ratio as a function of intra-binding site spacing *s* and looping length *N*. (**c**) Expression level ratio measurements for synthetic enhancers with a tandem of TraR-binding sites (blue) that are in-phase with fixed inter-TF spacing (*s*=23 bp); see schematic on the right. The single TraR-binding site synthetic enhancer data are overlayed as reference in red. (**d**) Model predictions for looping probability ratio as a function of distance to the chain origin (*k*) for the following configurations: blue, in phase tandem protrusions (*s*=21 bp); red, single protrusion. Error bars correspond to the s.d. from multiple measurements.

To do this, we constructed a second synthetic enhancer library with two TraR-binding sites. To determine the optimal binding-site arrangement for quenching, we first scanned the expression-level ratio values for a set of tandem TraR synthetic enhancers characterized by simultaneously varying values of the inter-site spacing *s* and the looping length *N* at 1 bp increments, while keeping *k* constant (Fig. 3a). We plot the results for the expression-level ratio as a function of the spacing *s* in Fig. 3a. The figure shows an oscillating function with a significantly stronger maximal quenching response (∼30%) observed for *s*=23 than the one observed for the single TraR-binding site (∼70%) and slightly larger maximal quenching than the response obtained for the single LacI-binding site. The figure also shows expression-level ratio minima at inter-site spacing values that are integer multiples of the helical repeat (that is, *s*=23–24, 34–35 and 44–45), whereas no quenching is observed for odd half-integer multiples of the spacing, as predicted by the model. Interestingly, the values of the expression-level ratio minima and maxima shift to higher values (from 30 to 60% and 80 to 100%, respectively) as the number of helical repeats between binding sites increases from two to three, to four. In Fig. 3b, we plot the model's predictions for the effects of inter-protrusion spacing (*s*) on the probability of looping. As in the experiment, the cross-section was taken for values of *s* and *N* that varied together by one-link increments for each successive point (Supplementary Fig. 3a dashed line), whereas the protrusion position (*k*) was kept constant at four helical repeats (42 links). Remarkably, the simulation exhibits not only oscillations in the probability ratio levels as expected but also an overall upward shift in the values of the probability ratio minima and maxima, in close agreement with the experimental data.

To further validate the volume additivity prediction, our second library also included synthetic enhancers with a tandem of TraR binding characterized by a fixed inter-site spacing (*s*=23 bp, in phase), while the placement of the proximal TraR-binding site from the (*k*) was varied. In Fig. 3c, we plot the expression-level ratio results (blue circles). The data stably oscillate from a strong quenching value of ∼40% to no-quenching or slight upregulation values of 100–110%. Although this behaviour persists for binding site positions that are spread over 100 bps, as *k* increases further so that the location of the tandem binding sites approaches the promoter, the amplitude of the oscillations diminishes and a clear bias towards upregulation emerges, with a maximal upregulation value of 160% observed for *k=*189 bp. The oscillatory pattern is highly repetitive with a periodicity of 10.5±0.3 bp, an expression-level ratio amplitude that is approximately twice as large as for the synthetic enhancer with a single TraR-binding site (Fig. 3c red x's) and persists for nearly the entire looping length (∼227 bp) with little dependence on the position of the first binding site.

Comparing the experimental data with one-dimensional cross-sections of the modelling results (Fig. 3d and Supplementary Fig. 3b dashed line) for thick chains with a single protrusion (red line) and a tandem of in-phase protrusions (*s*=21 links, blue line), the model captures the experimental trends nicely. Here, the in-phase tandems exhibit a probability ratio response, which is characterized by a significantly larger amplitude of oscillations, as compared with the chain containing a single protrusion. In addition, the in-phase tandems exhibit oscillations whose amplitude first increases as *k* varies from small values, reaches a maximum at *k∼N/*2, decreases for *k>N/*2 and increases again at *k∼N*, which is in agreement with similar trends observed experimentally.

Finally, we also varied the looping length *N* for in-phase (*s*=23 bp) and out-of-phase (*s*=28 bp) tandem TraR synthetic enhancers, while keeping the position inside the loop (*k*) constant, and once again found that experimental expression-level ratio measurements matched nicely with looping probability ratio predictions for the tandem TraR enhancer constructs (see Supplementary Fig. 4).

### Stiffening and bending effects

An anomaly observed in the experimental data for the tandem-TraR and LacI synthetic enhancers as compared with model predictions is the lack of significant upregulation in the former (except near the promoter for large values of *k*) and a complete absence thereof in the latter. We hypothesized that a bias towards quenching can emerge if the transcription factors also ‘stiffen' the DNA, making it slightly harder to bend locally. Based on our model, we expect such a bias to be dependent on the loop length (diminishing quickly for large *N*), the extent of the stiffened region (that is, the number of stiffening binding sites) and the binding sites' proximity to the centre of the loop (see Supplementary Notes 2 and 4 for additional discussion and Supplementary Fig. 5a,b).

**Figure 4:**
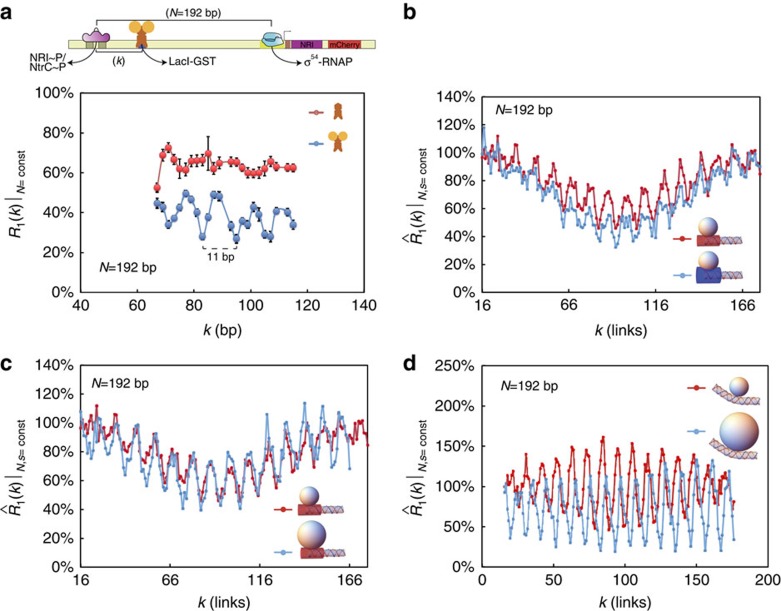
Combined elastic and entropic effects on looping. (**a**) Expression-level ratio measurements for the synthetic enhancers with a single LacI-binding site. Blue and red circles correspond LacI-GST and LacI expression-level ratios, respectively. (**b**–**d**) One-dimensional cross-sections for *N*=192 links comparing: (**b**) two values of stiffening with constant protrusion volume (blue) 2*b* and (red) 1.5*b*, where *b* is the DNA's Kuhn length (106 nm). (**c**) Two protrusion volumes (blue, 8.16 nm; red, 5.44 nm) at constant stiffening (1.5*b*). (**d**) Two protrusion volumes that can also bend the thick chain by 10° (blue, 8.16 nm; red, 5.44 nm). Error bars correspond to the s.d. from multiple measurements.

To experimentally test the validity of the stiffening-excluded volume-looping regulatory model and provide further support for the additivity finding, we fused the 25-kDa glutathione *S*-transferase (GST) domain to the carboxy terminus of LacI to make a new 63 kDa TF: LacI-GST. This allowed us to generate a significantly larger LacI (see Fig. 4a top schema), while not affecting its capacity to bind DNA (see Supplementary Methods). As both LacI (38 kDa, Fig. 2c) and a tandem of TraR proteins bound to two binding sites (2 × 25 kDa, Fig. 3c) seem to stiffen DNA as compared with a single bound TraR (25 kDa), we reasoned that a larger transcription factor may also add to the stiffening effect. Thus, according to the excluded volume portion of the model, the larger protein should generate a larger amplitude of oscillations between the regulatory minima and maxima, while the stiffening effect should shift the mean regulatory levels of these oscillations towards quenching.

To quantify the regulatory effect induced by LacI-GST when bound to the synthetic enhancers and compare with the effect generated by the native LacI, we measured the expression-level ratio for LacI-GST on the same synthetic enhancer library as the one used for LacI. The data are plotted in Fig. 4a. The figure shows that the expression-level ratio for LacI-GST (blue) exhibits an oscillatory function that varies from very strong quenching values (25–30%) to intermediate quenching (40–50%). The oscillations exhibit the 10.5-bp periodicity observed for the TraR tandems and the overall extent of the expression-level ratio indicates that LacI-GST generates significantly stronger mean quenching response (38%±7%) than the native LacI (64%±4%, red). Moreover, the amplitude of the oscillations that are observed for LacI-GST-bound synthetic enhancers (19%±4%) is approximately twice as large as the amplitude exhibited by the LacI synthetic enhancers (9%±5%). Finally, the LacI-GST expression-level ratio oscillatory function is phase flipped. Namely, the peaks of the LacI-GST data set appear at the minima of the LacI data set, and vice versa.

To compare the experimental results to the model, we plot in Fig. 4b–d three cross-sections. In Fig. 4b we show that by increasing the stiffness parameter (blue versus red line) the probability ratio gains an additional bias towards quenching. In Fig. 4c we show that for the same stiffness value, increasing the size of the protrusion by a small amount (blue versus red line) leads to an increase in the amplitude of the oscillations, as expected. However, to account for the phase flipping, another mechanism is needed. One possibility is bending, in which the transcription factor also ‘bends' the DNA locally (see Supplementary Notes 2 and 4 for additional discussion). We find (see Supplementary Fig. 5c,d) that when the bending protrusion is inside the loop, the probability of looping is upregulated, whereas when the protrusion is outside of the loop the probability of looping is reduced. Thus, to account for the phase flipping observed for LacI-GST as compared with LacI, we plot (Fig. 4d) the looping probability ratio for two scenarios. In the first, we simulate a thick chain with a small protrusion inside the loop that also bends the chain by 10° (red line). In the second, we simulate a thick chain with a 3 × larger protrusion positioned inside the loop, which bends the DNA by the same amount (blue line). The data show that for the thick chain with a smaller bending protrusion, the oscillations are consistent with a dominant bending effect generating an upregulation prediction for in-phase locations *k*. However, for the thick chain with the larger protrusion, the oscillations are consistent with a dominant excluded volume and stiffening effect generating a phase-flipped signal, which is similar to the one observed when comparing the LacI and LacI-GST synthetic enhancers.

### Deciphering higher-order TF-binding configurations

**Figure 5:**
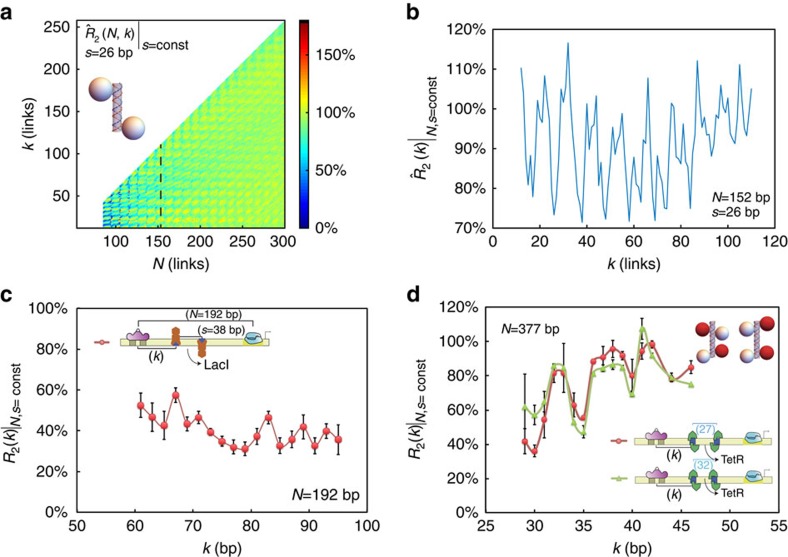
Periodicity of a half-helical repeat. (**a**) Heat-map depiction of the probability-ratio level for a thick chain with two ‘out-of-phase' protrusions taken at a fixed inter-site distance (*s*). Inset: chain schematic showing the Z-shaped binding architecture. (**b**) Cross-section of the heat map at *N*=152 links, showing oscillations with a 5- to 6-bp periodicity. Protrusion size was taken to be 5.44 nm in diameter. (**c**) Expression level ratio data for LacI synthetic enhancers with tandem binding sites spaced at 38 bp. (**d**) Expression level ratio data for synthetic enhancers with tandems of TetR-binding sites that are spaced by half integer (*s*=27 bp, red line) and integer multiples of the helical repeat (*s*=32 bp, green line), showing identical regulatory function. Inset: the structural binding model showing a thick chain with two dumbbell-shaped protrusions positioned in-phase (right) and out-of-phase (left), respectively. Error bars correspond to the s.d. from multiple measurements.

We next hypothesized that a pair of binding sites arranged in an out-of-phase configuration should generate a regulatory response with a periodicity that differs from the in-phase-tandems or single-binding-site cases. In Fig. 5a,b we plot model predictions for the regulatory output for an out-of-phase ‘Z-shaped' binding configuration showing that 5–6 bp oscillations are generated with a pattern of alternating strong and weak maxima/minima. This periodicity is a result of the fact that a 180° rotation of the Z-shaped tandem around the thick chain axis yields a similar configuration. The deviation between these two configurations is responsible for the alternating extrema. Although the overall regulatory effect predicted by the model is relatively small for such a configuration, for larger TFs a detectable signature may be observed.

We constructed a final synthetic enhancer library to test the 5–6 bp periodicity and alternating weak/strong extrema predictions for synthetic enhancers bound by Z-shaped TF structures. First, we characterized 18 synthetic enhancers with tandems of LacI-binding sites whose centre-to-centre spacing was set at 38 bp. Such an arrangement not only places the LacI dimers in opposite orientation, but also strongly restricts their ability to tetramerize. The binding sites' positions inside the loop were shifted together in 2 bp increments, thus covering a range of 36 bp of intra-loop positions. In Fig. 5c (red) we plot the results. The data show that the tandem-LacI synthetic enhancer strains exhibit a fluctuating regulatory response with a distinct 4–6 bp periodicity for the majority of intra-loop positions of the tandems and a slight increase in the overall magnitude of quenching as the binding sites are moved towards the centre of the loop.

Given these results and Z-shape model predictions, we wondered whether there was something amiss with our interpretation of the single binding-site expression-level ratio results for the TetR synthetic enhancer shown in Fig. 2d. This data set shows a strong regulatory response with sharp fluctuations between quenching and upregulation despite the fact that TetR is a small protein (25 kDa^42^), which is nearly the size of TraR (26 kDa^43^). In addition, a closer look at the expression-level ratio scan (1 bp increments) reveals that the oscillations do not exhibit the ∼11-bp periodicity expected from a single binding-site synthetic enhancer. Rather a complex pattern of strong peak/weak trough–weak peak/strong trough seems to emerge with a 5- to 6-bp periodicity between adjacent peaks and an 11-bp periodicity between ‘strong' peaks or troughs is also apparent. These results are reminiscent of the model predictions shown in Fig. 5a,b for the out-of-phase tandems and match nicely with a slightly more complex Z-binding configuration as shown in Supplementary Fig. 6b,c (orange line).

Consequently, to account for the periodicity and size effect anomalies, we hypothesized that our form of TetR (TetR-B^44^) might bind its binding site not as a dimer but rather as a dimer-of-dimers oriented in dumbbell-like configuration. This binding architecture is known for a member of the TetR family QacR^36^ and in this interpretation an additional cryptic binding site overlaps the major site, allowing a dumbbell-like bound TetR structure to form. To test our hypothesis, the second part of our final library was designed with synthetic enhancers containing tandems of TetR-binding sites. We designed two sets: (i) first, with the TetR-binding sites in-phase (*s*=32 bp) and (ii) second, with the binding sites out-of-phase (*s*=27 bp). If the dimer-of-dimer structural interpretation was correct, then the expression-level ratio for both binding site configurations should be nearly identical. This can be seen from a schematic of a thick chain with protrusions (Fig. 5d inset). In both configurations, two dimer-of-dimer protrusion structures are shown on the thick chain with an overall ‘out-of-phase' arrangement of two dimers inside the loop and two outside for the chosen inter-site spacings *s*.

In Fig. 5d we plot the expression level ratio measured as a function of *k* for these synthetic enhancers (*N*=377 bp), with green triangles and red circles for the in-phase and out-of-phase inter-site spacing configurations, respectively. The figure shows that the expression level ratio regulatory response generated by both tandems is nearly identical as predicted by the model, with a distinct 5–6 bp periodicity over a range of values for (*k*) that spans 20 bp at a single base-pair resolution. The regulatory pattern for both cases exhibits three distinct peaks and four troughs with a slightly increasing overall expression level ratio trend. In addition, the data sets lack the strong peak–weak peak pattern of the single binding-site synthetic enhancer. As a result, the dimer-of-dimer dumbbell binding structure for TetR may indeed be a possibility *in vivo*, despite not having been observed in classic *in vitro* experiments (see Supplementary Fig. 6d).

### INDEL mutations in natural bacterial enhancers

Finally, we wondered whether we could find evidence for the excluded-volume regulatory model in bacterial genomes. As this type of regulation depends on a fixed relative arrangement of the self-avoiding volumes, we speculated that naturally occurring enhancers should exhibit a conserved evolutionary signature for this mechanism if it does indeed play a biological role. Specifically, we speculated that bacterial enhancers with similar regulatory function should be insensitive to ∼11 bp INDEL mutations that conserve both the regulatory TF and activator orientations relative to the promoter and sensitive to function-altering INDELs that are 5 or 6 bp long. As a result, we expected that looping sequences should reflect this tolerance to the DNA helical repeat.

**Figure 6:**
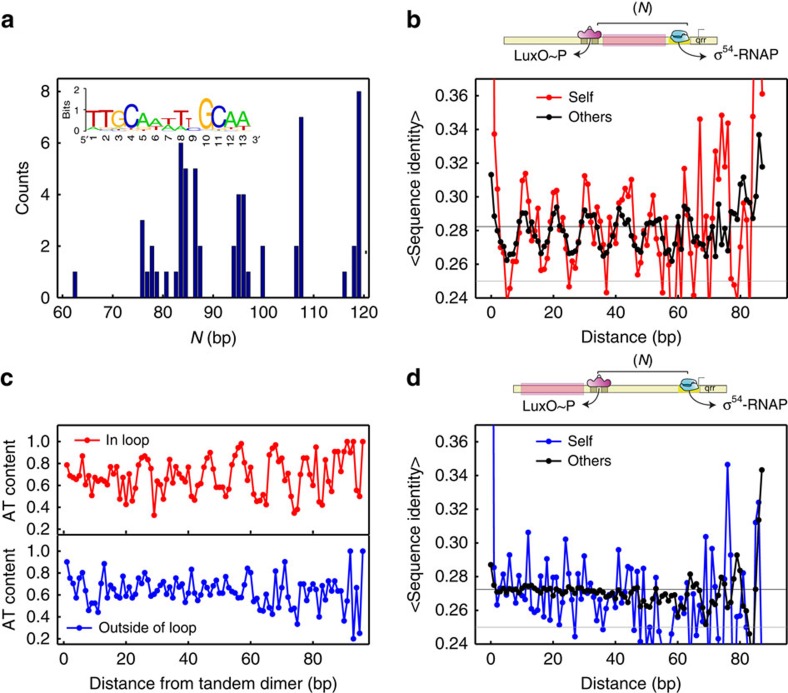
Sensitivity to the DNA's helical repeat in the *Vibrio qrr* enhancers. (**a**) Histogram of loop lengths for all putative loop sequences. Inset, LuxO consensus sequence. (**b**) Relative identity of all putative loop sequences to themselves (red) and to all other putative loop sequences (black). Horizontal lines indicate the expected identity level for sequences with equal probability for all four nucleotides (grey) and for the putative loop sequences (black) (see the Supplementary Methods). (**c**) AT nucleotide content as function of position for the loop sequences (top, red) and for the non-looping upstream sequences (bottom, blue). (**d**) Relative identity for all non-looping upstream sequences (from the tandem LuxO binding sites, see top schematic) to themselves (blue) and to all other upstream sequences (black).

To test this hypothesis, we analysed the *qrr* (quorum regulatory RNA) genes in the *Vibrio* genus. Some of the *qrr* genes in this genus are known to be regulated by LuxO, an NtrC-like activator, which drives σ^54^ promoters. This system was implicated in the quorum-sensing pathway and was characterized for *Vibrio cholerae*^45^,^46^. Using standard bioinformatic tools (see Supplementary Methods), we annotated 61 *qrr* enhancers (see Supplementary Data 2), which included a putative LuxO-binding site tandem (see Fig. 6a (inset) for consensus sequence and also Supplementary Table 1), a looping region and a single putative σ^54^ promoter (Supplementary Fig. 7). In Fig. 6a we plot the distribution of the looping lengths for all 61 putative *qrr* enhancers. The figure shows a set of clustered looping lengths (*N)* ranging from an average length of ∼80 to ∼120 bp, which are displaced from one another by ∼11 bp, thus providing initial support for our hypothesis.

To further test the sensitivity of the *qrr* looping regions to integer multiples of the helical repeat, we checked the average identities of each loop sequence to itself and to all other loop sequences. To do this, we calculated the relative identity of each 9 bp window within a given loop sequence to all other 9 bp windows either on the same sequence, or on all other sequences, noting the distance between the positions of the first bases of compared windows (the relative identity is defined as the number of positions (that is, from 0 to 9) for which both windows contain the same base, divided by the window length). We then computed the mean relative identity for each window separation by averaging over all relative identities in a particular window-separation value. In Fig. 6b we plot the results. The figure shows that the mean relative identity for the annotated *qrr* enhancers exhibits an oscillatory behaviour, which persists for all possible values of the distance between the windows. Interestingly, the oscillatory pattern is detected not only for cross-correlated enhancers, but also within each enhancer to itself (self, red; other, black), with the first maxima appearing at ∼0 bp displacement and with periodicity of 10.45 bp.

Next, we checked whether there was some underlying signature for a conserved sequence within the looping region. To that end, we computed the average AT/GC content of each position within the loop and plotted the results in Fig. 6c (top). The figure shows that the AT content is enriched at positions that are integer multiples of 10.6 bp with at least six distinct peaks visible in the data. In addition, the minima between the positions of AT enrichment converge on a content value of ∼0.5, which is the value expected for a random allocation of AT or GC at those particular positions. Thus, loop sequences are similar either at the same relative position or alternatively at positions displaced by an integer multiple of the helical repeat from the position of the reference sequence, with a preference to AT segments.

Finally, in Fig. 6c (bottom) we plot the average AT/GC content and in Fig. 6d the average relative identities of the *qrr* enhancer ‘upstream sequences' that are immediately adjacent to the annotated LuxO-binding site tandem (Fig. 6d schematic). Unlike the looping region, the analysis on the non-looping region results in no particular repetitive pattern of AT/GC content within the upstream sequence. In addition, a monotonic or slightly varying signal across all possible values of the window displacement is observed in the upstream region without any detectable characteristic oscillations. Thus, the striking difference between Fig. 6b,d and in Fig. 6c (red versus blue line) provides further support to the special sensitivity of *qrr* enhancer sequences to the helical periodicity as compared with non-looping sequences.

## Discussion

We presented a new mechanism for quenching-like repression in bacterial enhancers using a combined thermodynamic modelling, synthetic biology and bioinformatic approach. First, we constructed a preliminary DNA looping-based mechanistic structure–function model. The model established a direct relationship between the values of enhancer structural control parameters (for example, number of binding sites for a TF, inter-site spacing, looping length and so on) to a predicted regulatory function that was based on elastic (bending and stiffening) and entropic (excluded volume) characteristics of a thick chain with protrusions. Second, based on the numerical results we characterized synthetic enhancer libraries focused on testing the predictions of the structure–function model, allowing us to improve the mechanistic model. Finally, we provided biological validation for the findings of the model and synthetic enhancer experiments using bioinformatic predictions on a multitude of naturally occurring enhancers. Altogether, we needed only 303 synthetic bacterial enhancers to mechanistically characterize the excluded-volume quenching mechanism, showing that our model-based design and experimentation approach can achieve insight into a natural phenomenon with a much smaller data set as compared with conventional high-throughput methodologies.

To fully account for all of our experimental observations, we had to incorporate small local bending and stiffening effects into our excluded-volume model. Interestingly, these additional elastic effects, which fine-tuned our model, have made both our experiments and model applicable to past observations made on bacterial enhancers. These include studies of IHF-dependent enhancers^9^,^10^, where IHF was shown to both upregulate and downregulate expression depending on position^29^, and non-IHF-dependent enhancers^11^,^12^,^13^. In these studies, the ‘regulator' binding sites were typically located halfway between the promoter and activator (consistent with our prediction that the maximal regulatory effect should occur near the centre of the loop, see Supplementary Fig. 2 and associated discussion), and the regulatory effect depended strongly on whether the DNA-binding protein was positioned inside or outside the loop. As a result, a novelty of our experimental and modelling results is that the conservation of TF-binding orientation within bacterial enhancers seems to be a generic phenomenon for all transcription factors and is not limited to a handful of DNA-bending proteins (see also Supplementary Table 2 and Supplementary Note 4). Finally, although our bioinformatic observation is consistent with this conclusion, our model and computational analysis cannot decipher whether conserved sequences in natural bacterial enhancers correspond to TF-binding sites or to other sequences with special physical characteristics (for example, AT-rich curved segments).

An additional implication of our results is that complex binding arrangements of several transcription factors within an enhancer should generate unique regulatory signatures. Such a signature was predicted and observed for the Z-shaped binding arrangement for the anti-phase LacI synthetic enhancers, exhibiting a regulatory signature with a 4–6 bp periodicity. Building on this observation, we used the results from our assay and model to propose that TetR-B may actually bind our synthetic enhancers *in vivo* as dumbbell-like dimer-of-dimer structure in a similar manner to its homologue QacR^47^. This interpretation of our data was based on two observations: the first was an anomalously large regulatory effect as compared with TetR's size (25 kDa∼TraR) and the second was the 5- to 6-bp periodicity observed for the single TetR and for all the tandem TetR synthetic enhancer binding-site architectures. However, our inability to provide further support for this structural interpretation using our own or past^48^ *in vitro* gel-shift experiments indicates that there are still many unresolved issues with our understanding of protein–DNA interactions *in vivo.*

Finally, there are broad genomic implications to our results. As excluded volume can affect the behaviour of polymers in the entropic regime (implying that it may be applicable to long-range interactions, unlike local bending and stiffening), the conservation of relative binding orientation between adjacently bound transcription factors may not only be relevant to bacterial enhancers or promoter-proximal regulatory regions in eukaryotes, but also to eukaryotic enhancers as well. Owing to the sensitivity of our model to boundary conditions, applying it to such systems will have to take into account molecular details to properly set the looping criterion, as was done here for the bacterial σ^54^ promoter. At the very least, our experimental, modelling and bioinformatic analysis suggest that sensitivity to INDELs that are integer multiples of the helical repeat could be an evolutionary fingerprint for enhancers, whereas INDELs of odd half-integer multiples of the helical repeat should be flagged as candidates for important regulatory variation.

## Methods

### Synthetic enhancer cassette design

Synthetic enhancer cassettes (see Supplementary Table 3) were ordered as double-stranded DNA minigenes from Gen9 Inc. Each minigene ordered was ∼500 bp long and contained the following parts: BamHI restriction site, tandem NRI-binding sites from glnAp2 promoter (also containing the σ^70^ glnAp1 promoter), the σ^54^ glnAp2 promoter and a HindIII restriction site. In addition, each minigene contained a looping segment in between the NRI tandem binding sites and the σ^54^ promoter. The looping segment was of variable length (*N*) and contained either one or two binding sites for TraR, TetR or LacI. The binding sites were positioned in varying inter-site spacings (*s*) from one another and locations away from the NRI binding sites (*k*) within the looping region. For insertion into synthetic enhancer plasmids, minigene cassettes were first double-digested with BamHI/HindIII before being used as an insert in the cloning step. Cloning was then carried out into a basic template synthetic enhancer plasmid as previously described^8^,^49^. Briefly, synthetic enhancer sequences were computationally designed to have a minimal probability to bind DNA-binding proteins. This was done by constructing an algorithm that randomly generated a set of sequences, which were compared with the roughly 2,000 known specific DNA-binding sites for *E. coli* transcription factors obtained from RegulonDB (http://regulondb.ccg.unam.mx). Sequences were design with 40–50% AT/GC content (see Supplementary Data 1).

### Strain construction

The synthetic enhancer strains were constructed as described by Amit *et al.*^8^,^49^. Briefly, *E. coli* strain 3.300LG^50^ with deletions for *glnL* and *glnG* genes was transformed with sequence-verified pACT and synthetic enhancer plasmids. The pACT family of plasmids was constructed by modifying p3Y15 (ref. 50). We inserted a *lacI* gene and either a *tetR* or *traR* gene into the parent plasmid, under the control of the same glnL promoter controlling the NRII2302 mutant. The tetR sequence that we used is that of TetR-B^44^, which we refer to as TetR. Selection was carried out via double Kan/Amp resistance (20 and 100 mg ml^−1^, respectively). Candidate synthetic enhancer strains were tested for fluorescence in the presence and absence of the suitable inducer (see below) on a plate reader (Tecan, Infinite F200), to ensure that a proper strain was constructed. All synthetic enhancer sequences can be found in Supplementary Data 1.

### Expression level ratio measurement assay

Expression level measurements for all synthetic enhancers without LacI-binding sites were carried out as follows: first, synthetic enhancer strains were grown in fresh Luria-Bertani with appropriate antibiotics (Kan/Amp) to midlog range (OD_600_ of ∼0.6) as measured by a spectrophotometer (Novaspec III, Amersham Biosciences) and were resuspended in low-growth/low-autofluorescence bioassay buffer (for 1 litre: 0.5 g Tryptone (Bacto), 0.3 ml glycerol, 5.8 g NaCl, 50 ml of 1 M MgSO_4_, 1 ml 10 × PBS buffer at pH7.4 and 950 ml double distilled water). Isopropyl β-D-1-thiogalactopyranoside (IPTG; 1 mM) was added at this point, to deactivate the LacI protein that represses the glnAp2 promoter in the pACT plasmid. Two millilitres of resuspended culture with IPTG were dispensed into each well of a 48-well plate. Appropriate concentrations of anhydrotetracycline (aTc, Cayman Chemical 10009542) or *N*-(3-Oxooctanoyl)-L-homoserine lactone (3OC8, Sigma-Aldrich O1764) were dispensed into each well, spanning four to six orders of magnitude. Up to 24 levels of aTc or 3OC8 concentration were used for each strain. The plates were then incubated in a 37 °C shaker until cultures reached steady-state growth. Measurements of fluorescence levels were taken by dispensing 200 μl of culture into each well of a 96-well plate and were carried out on a plate reader (Tecan F200). Two wells were used as IPTG controls. We carried out each measurement in duplicate. All fluorescence measurements were divided by optical density, to measure the normalized expression, and autofluorescence levels (cells with no plasmid) were subtracted from the normalized values. Some TetR and TraR synthetic enhancers were also tested in strains lacking the LacI protein and no distinguishable difference in the regulatory response was observed.

### LacI experiments

Synthetic enhancer cassettes containing LacI-binding sites were cloned into a similar template plasmid as other cassettes, containing glnAp2 promoter and two NRI-binding sites, except the LacI-binding site was removed from the glnAp2 promoter. The removal of the exogenous LacI sites were done to ensure that no tetramerization could take place between the site on the enhancer and these sites, thus affecting the regulatory outcome. The cassettes were cloned into 3.300LG strain along with pACT plasmid (expressing LacI). The experiment was carried out as described above using IPTG as an inducer for LacI removal from the synthetic enhancer-binding site.

### Expression level ratio and probability of looping ratio

To compute the expression level ratio *R*_1_(*N,k*) for a synthetic enhancer (see Fig. 1c bottom schematic and Supplementary Equation (39) for precise definition), we take the ratio in fluorescence expression levels between the protein-unbound regime to the protein-bound case for each measurement of a synthetic enhancer's regulatory response curve. Typical regulatory-response curves for individual synthetic enhancers are shown in Supplementary Fig. 8. For example, in Supplementary Fig. 8a–f we plot data sets obtained for two TetR, one LacI and three TraR synthetic enhancers, showing sigmoidal transfer functions when varying the concentration of aTc, IPTG and 3OC8 from low values to saturating levels, respectively. All expression level ratio measurements in our experiments were obtained using such a procedure with the protein-unbound case regimes set at low 3OC8, high aTc and high IPTG for TraR, TetR and LacI, respectively.

### Error estimation

We employed two distinct error estimation methods, which both yielded a 3–7% estimated error in expression-level ratios. Variance in expression-level ratio measurements was calculated as follows: multiple (≥) measurements were taken at 30-min intervals for each of the 2 true duplicates of each strain at 24 inducer concentrations. For the raw data shown in Supplementary Fig. 8, the multiple measurements of duplicates were averaged and the s.d. of the measurements was used as the error bars.

For the expression-level ratio, two concentration ranges in which the fluorescence data levels remained constant were selected for each strain. For instance, in Supplementary Fig. 8a,b we chose the low aTc concentration range (in which the DNA is highly likely to be bound by a TF) and high aTc concentration range (in which the DNA is highly unlikely to be bound by a TF). For each of the two duplicates (1,2), the data in both ranges were averaged, yielding mean_*i,l*_ and mean_*i,h*_, respectively, where *i*∈[1,2] denotes the duplicate index. The expression-level ratio for each duplicate was then obtained by 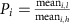. The final expression-level ratio was obtained by averaging the two expression level ratios *P*_1_, *P*_2_. The expression-level ratio error was obtained using error propagation analysis on the variance in the different mean values as follows: 
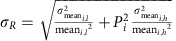
.

To verify the error estimate and expression-level ratio estimates obtained by the previous method, we employed a Hill function fit analysis. Here we took fluorescence level measurements as a function of inducer concentration (for example, Supplementary Fig. 8d,e) and fitted this data with a Hill function of order 1. This allowed us to determine the fluorescence expression levels for the occupied and unoccupied enhancer states from the Hill function fit parameters. The ratio of those two numbers was taken to be the expression level ratio. Error bars were determined by taking into account the errors in the hill function fitting parameters. The results from this analysis were indistinguishable within experimental error to the resulting from the alternative error analysis.

### Simulation parameters

In all of our simulations for the synthetic-enhancer-based regulation, we used the following parameters for DNA: Kuhn length *b*=100 nm (ref. 51), bending constant *a*=*b*/2, chain link length of *l*=0.34 nm (corresponding to a single base pair of the DNA), width of *w*=4.6 nm (ref. 52), 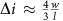 (see Supplementary Equation (13)), a helical repeat of *P*=10.5 bp (ref. 53) and a twisting constant of *c=*294 (corresponding to 100 nm)^51^ (Supplementary Equation (2)). Our choice for the value for *Δi* was made, on the one hand, to be reasonably low, to allow the hard-wall potentials to interact at as close a distance as possible, whereas on the other hand we verified that it fell within a range of values where the simulation was not sensitive to the particular choice of this parameter (data not included). The radius of the corresponding protrusion is ≈3.5 nm (refs 3, 54) and the polymerase protrusion radius is ≈7.2 nm (ref. 55). The TF protrusion radius is ≈2.72 nm (corresponding to a TetR transcription factor)^56^ unless stated otherwise. We also used the following parameters for the looping boundary conditions (see Supplementary Note 2 section 2.3.1): *ɛ*=8.4 nm, *δω*=90°.

### Robot measurements

High-resolution experiments were performed on a Tecan EVO 100 MCA 96 multichannel liquid handling system. Experiments were run as described above with slight changes: cells were grown to OD_600_ of ∼0.1 and 200 μl volume in a 96-well plate, centrifuged and resuspended in bioassay buffer. Inducers were added manually to an inducer plate and then spread in different concentrations automatically to the 96 wells. Fluorescence measurements were taken using a plate reader (Tecan, F200) every 30 min.

### Fusion proteins construction

Fusion proteins were designed by connecting the GST coding sequence to either the C or N terminus of TraR, N terminus of TetR and C terminus of LacI coding sequences. GST was PCR amplified from PGEX-4T1 and cloned into pACT, pACT-TetR or pAct-TraR plasmid using Gibson assembly^57^. His-GST-TetR fusion was created by cloning GST-TetR into PGEX-4T. Fusions were sequence verified and their expression was verified by Coomassie or western blot analysis.

### Simulation and bioinformatic code

All computer code used in this work either for the numerical simulations or bioinformatic analysis can be made available on request.

## Additional information

**How to cite this article:** Brunwasser-Meirom, M. *et al.* Using synthetic bacterial enhancers to reveal a looping-based mechanism for quenching-like repression. *Nat. Commun.* 7:10407 doi: 10.1038/ncomms10407 (2016).

## Supplementary Material

Supplementary Information Supplementary Figures 1-8, Supplementary Tables 1-2, Supplementary Notes 1-4, Supplementary Methods and Supplementary References.

Supplementary Data 1 Supplementary Data 1 includes all the DNA cassettes sequences used in the manuscript along with their GC content.

Supplementary Data 2 Supplementary Data 2 describes the Vibrio qrr loop sequences used in our bioinformatic analysis. The first tab summarizes how the sequences were chosen. The second tab contains the nucleotide sequences used in the final analysis.
