# Supplementary material for: Using synthetic bacterial enhancers to reveal a looping-based mechanism for quenching-like repression
Source: Nat Commun. 2016 Feb 2;7:10407. doi: 10.1038/ncomms10407 (PMC4740811; doi:10.1038/ncomms10407)
Supplement: Supplementary Information — Supplementary Figures 1-8, Supplementary Tables 1-2, Supplementary Notes 1-4, Supplementary Methods and Supplementary References. [file ncomms10407-s1.pdf]

Supplementary Information

Contents

1 Supplementary Figures **3**

2 Supplementary Tables **11**

3 Supplementary Note 1: looping in the context of the wormlike chain (WLC) model **12**

3.1 The discrete WLC model 12

3.2 The discrete WLC Monte-Carlo algorithm 13

4 Supplementary Note 2: looping in the context of the self-avoiding wormlike chain (SAWLC) model **13**

4.1 The discrete SAWLC model 13

4.2 The discrete SAWLC Monte-Carlo algorithm 14

4.3 Protein-DNA interactions and looping in the context of the SAWLC model 15

4.3.1 Modeling a DNA chain with a bound protein 15

4.3.2 Monte-Carlo simulation of a DNA chain with a bound protein 15

4.3.3 Modeling bending and stiffening of a thick chain 15

4.3.4 Looping criterion 16

4.3.5 Modeling looping of a thick chain with a bending or stiffening protrusion . . 17

5 Supplementary Note 3: modeling the experiment **17**

5.1 Thermodynamic modeling of looping-initiated transcription 17

5.2 Connecting thermodynamic model and experiment 20

5.3 Enhancer with transcription factor (TF) binding site 21

5.4 Enhancer with two TF binding sites 21

6 Supplementary Note 4: supporting data **22**

6.1 Supplementary model data for Figure 1: A geometric model for a maximal regulatory effect at the $k = N/2$ position 22

6.2 Supplementary model data for Figure 3: a thick chain with two protrusions 23

6.3 Supplementary experimental data for Figure 3: tandem TraR synthetic enhancers with varying looping length and constant s 24

6.4 Supplementary model data for Figure 4 24

6.4.1 Combined excluded volume and stiffening 24

6.4.2 Combined excluded volume and bending 25

6.5 Supplementary model and experimental data for Figure 5 25

6.5.1 Experimental results and modeling: LacI-GST out-of-phase tandems 25

6.5.2 Experimental results: gel shift assay for TetR 26

6.6 Supplementary bioinformatics data for Figure 6: pAstC/pAruC promoters 26

7 Supplementary Methods **27**
7.1 Protein expression and purification 27
7.2 Electrophoretic mobility shift assays 27
7.3 The *Vibrio* genus *qrr* genes 27
7.3.1 Annotating the *qrr* σ^{54} promoters 27
7.3.2 Extracting the LuxO binding sites 28
7.3.3 AT/GC variation within the *qrr* enhancer sequences 29
7.4 Testing functionality of fusion proteins 29
7.4.1 TraR fusions 29
7.4.2 LacI fusions 29
7.4.3 TetR fusions 30

Supplementary References **31**

1 Supplementary Figures

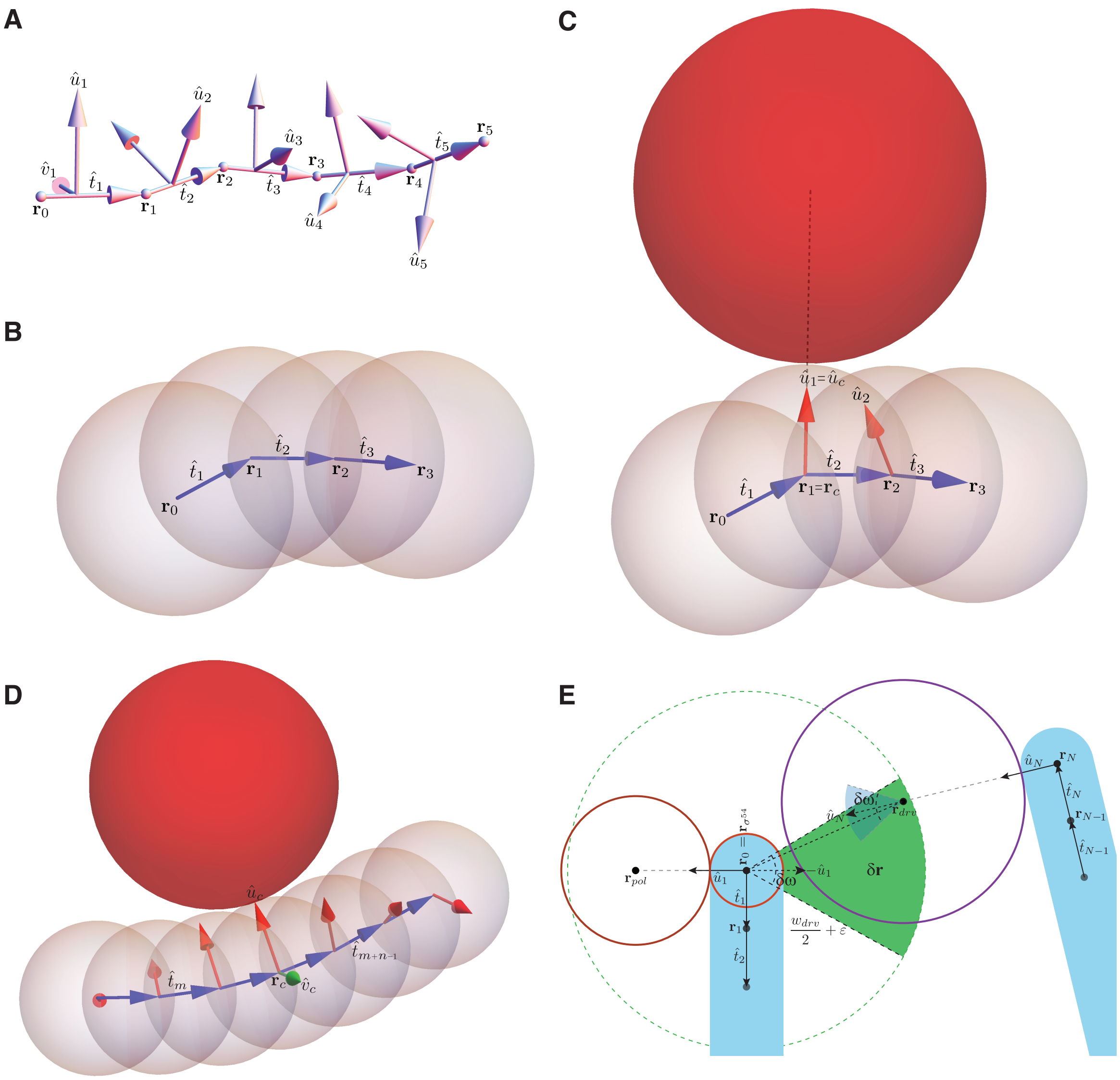

Supplementary Figure 1: Discrete WLC and SAWLC numerical algorithm schematics. (A) An example of a WLC with 5 links and $l = 1$. (B) An example of a SAWLC with $w = 2.5l$. (C) An example of a SAWLC with $w = 2.5l$ and a protrusion with diameter $w_{TF} = 4l$ (red sphere) located at the first link. (D) An example of a SAWLC with $w = 2.5l$ with a protrusion with diameter $w_{TF} = 4l$ (red sphere) bending the chain by 30°. (E) Illustration (not to scale) of driver-polymerase interaction criteria. Both proteins are bound to the DNA (light-blue cylinders) and directed along $\hat{u}$ from the links' centers. The driver (purple circle) is bound to the last link and the polymerase (red circle) to the first link, which is also identified as the location of the σ^{54} factor. The centers of the driver and the polymerase are denoted by r_{drv} and r_{pol}, respectively, and the center of the σ^{54} factor is denoted by $r_0 = r_{\sigma^{54}}$. The interaction volume δr in which r_{drv} can reside is shown as a light-green wedge. The possible directions for $\hat{u}_N$ are shown as a light-blue cone with its base in r_{drv}.

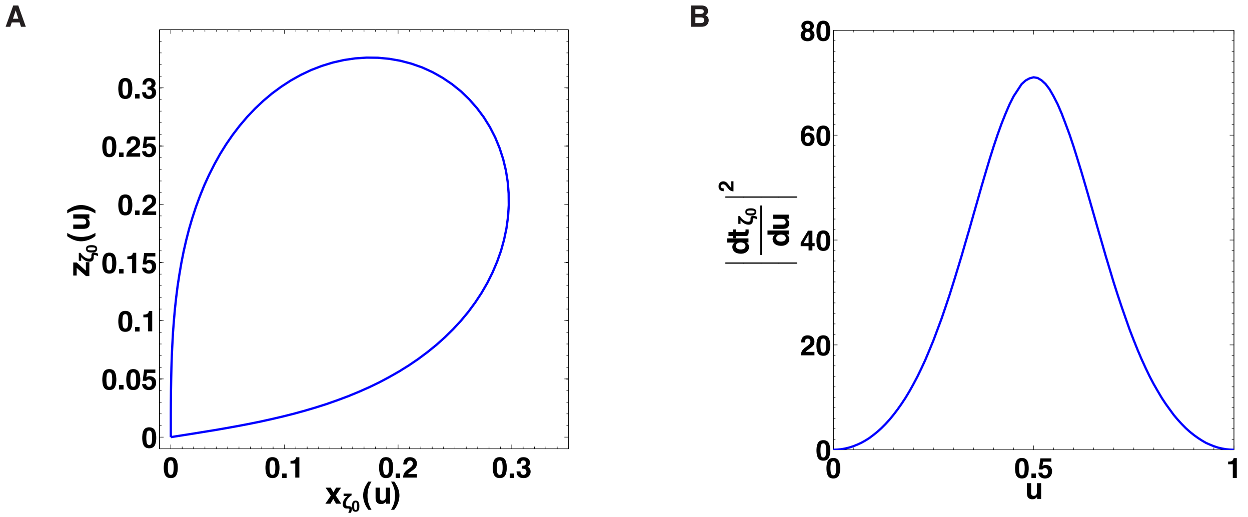

Supplementary Figure 2: Most-probable loop configuration and curvature analysis. (A) The most probable conformation $\mathbf{r}_{\zeta_0}(u)$ for a wormlike chain**.** (B) The squared curvature of the most probable conformation $\left|\frac{d\mathbf{t}_{\zeta_0}(u)}{du}\right|^2$ for a wormlike chain.

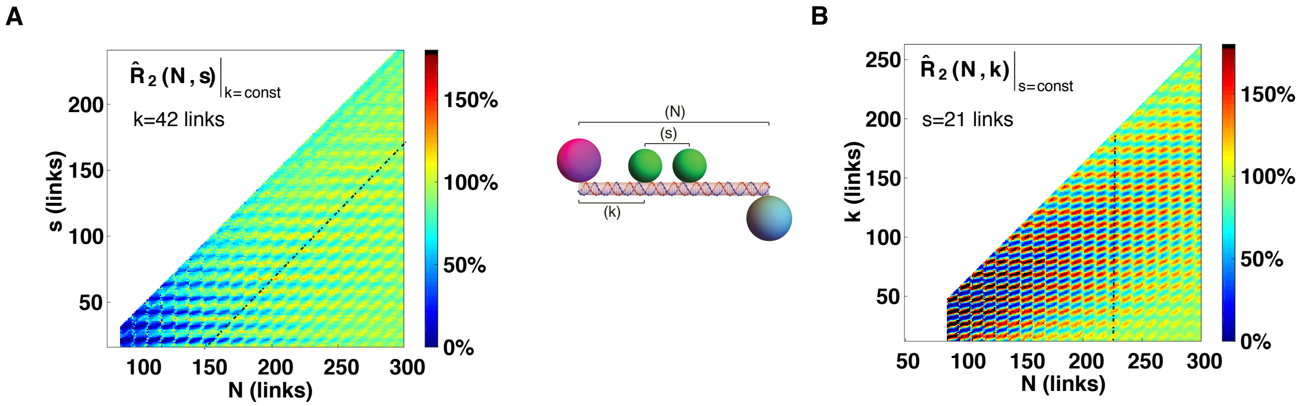

Supplementary Figure 3: 2D heat map cross-sections of the (N, s, k) 3D model for a thick chain with two protrusions. (A) N vs. s heat map for a tandem protrusion configuration with varying inter-site separation, and set at a constant distance from the chain origin ($k = 42$ bp). (B) N vs. k heat map for "in-phase" tandem configuration of protrusions ($s = 21$ links). (Inset) Schematic showing a small portion of the thick chain with protrusions. Protrusion size was taken to be 5.44 nm in diameter.

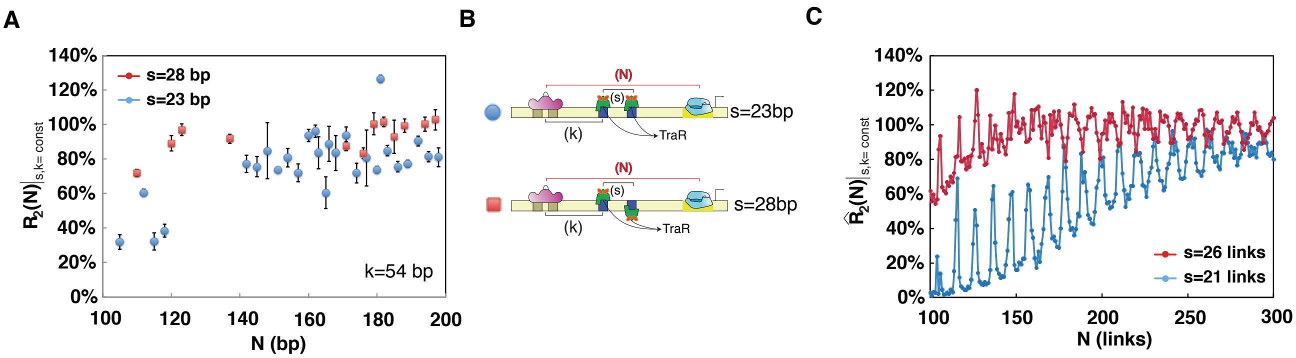

Supplementary Figure 4: Looping length variation: measurement and model. (A) Data showing the expression level ratio for a tandem of TraR binding sites with spacing $s = 23$ bp (blue) and $s = 28$ bp (red) with constant location k of the first binding site. The looping length N is varied in 2-3 bp jumps. (B) Schematic of the two constructs showing the $s = 23$ in-phase orientation (top), and $s = 28$ bp out-of-phase orientation (bottom) . (C) 1D contour maps of (N, s, k) probability ratio level space taken at $k = 53$ links, with $s = 21$ links (blue) and $s = 26$ links (red).

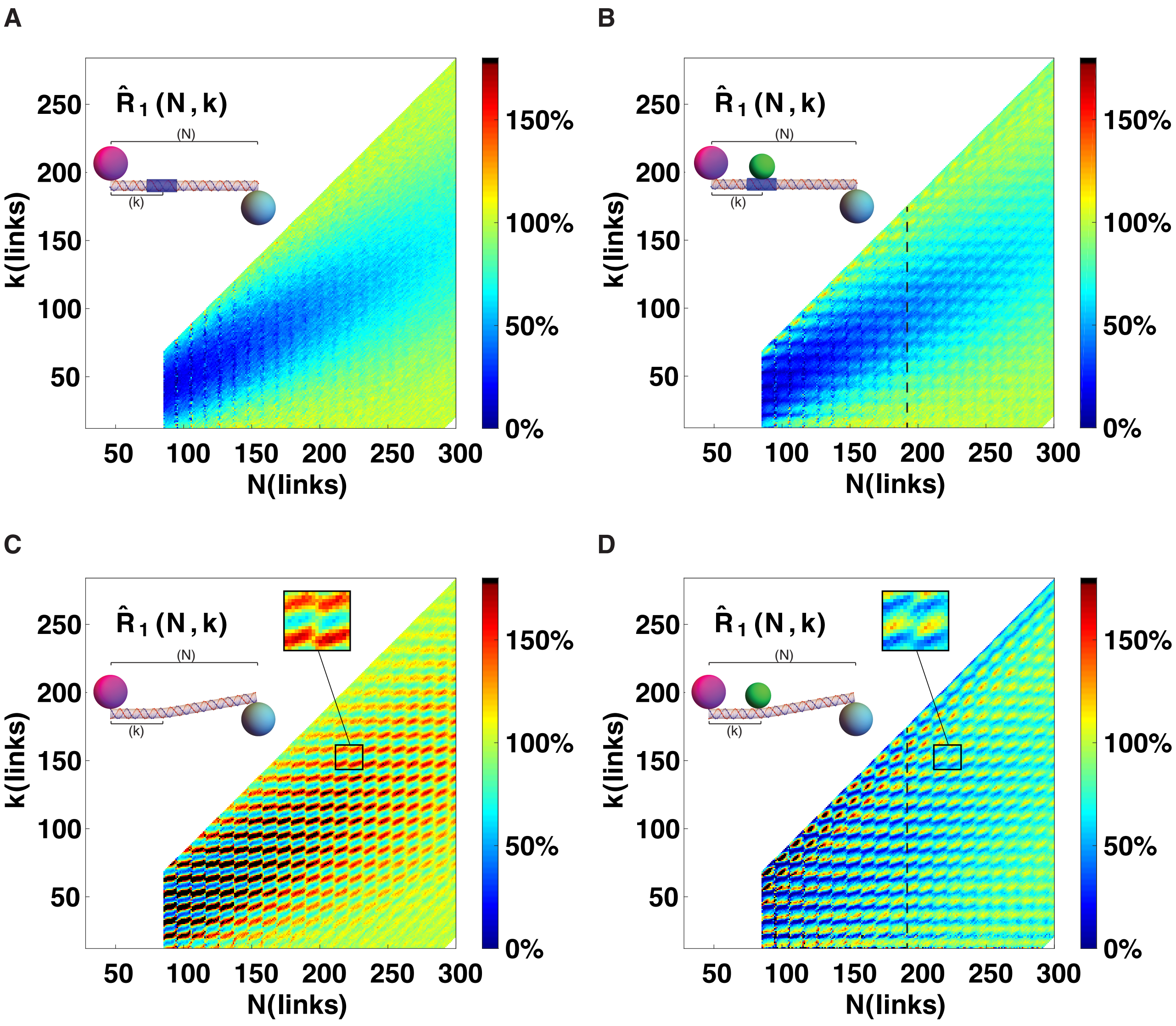

Supplementary Figure 5: Thick chain models with additional elastic deformations. (A) Wormlike chain model with stiffening. (B) Self-avoiding wormlike chain model with a protrusion that induces stiffening of $2a$ (factor of 2). (C) Self avoiding worm-like chain with a bender that has zero volume. (D) Self-avoiding wormlike chain with a protrusion of radius 3x2.72 nm which can also bend DNA. The bend in panels (C) and (D) is $10°/16$ per link, over 16 links. Insets in (C),(D) are over the same range of [210,230]×[145,160] in $N \times k$.

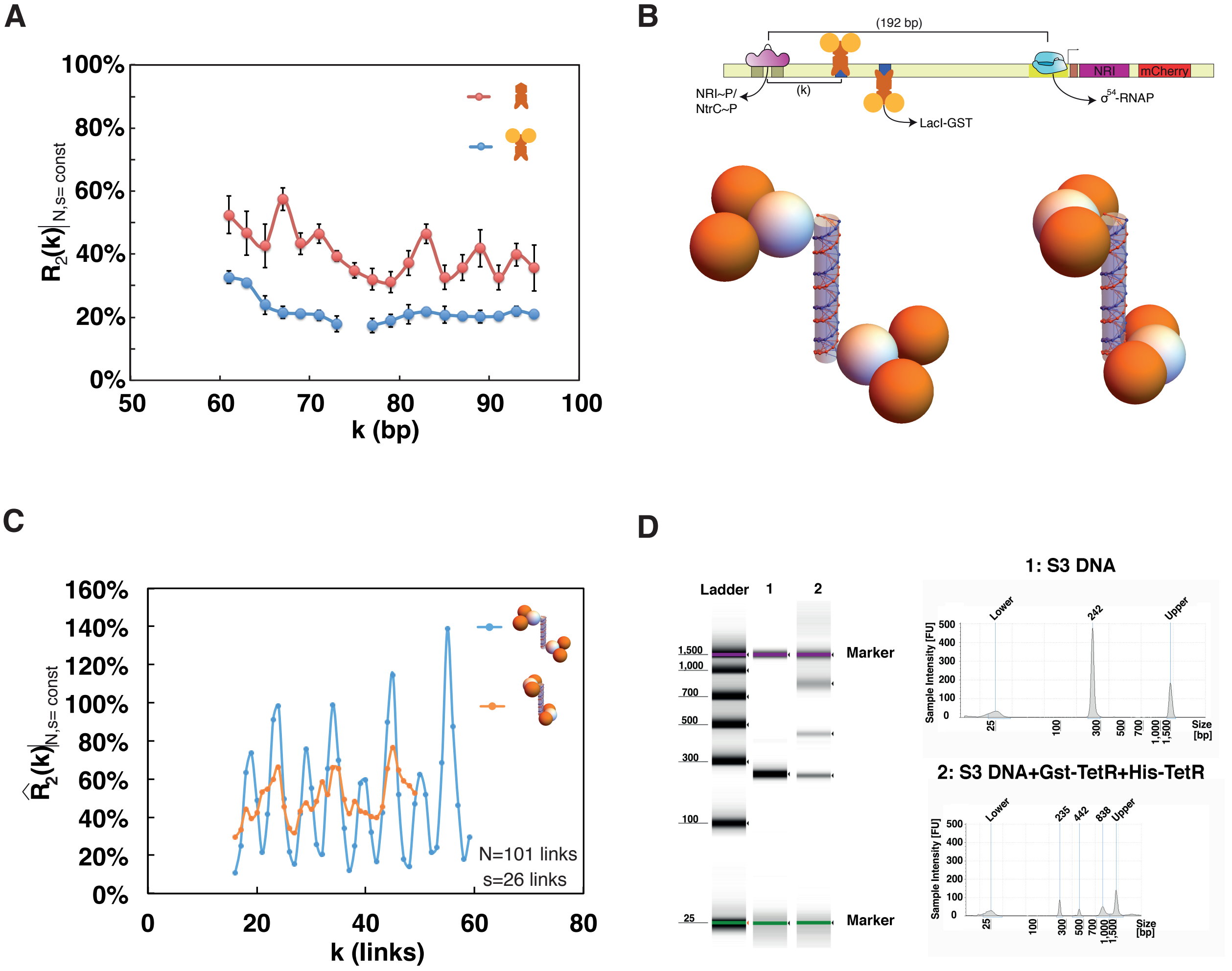

Supplementary Figure 6: Z-shaped structures and analysis of TetR-GST. (A) Synthetic enhancer with LacI binding sites positioned in Z-shaped orientations. Expression level ratio for LacI (red) and LacI-GST (blue) showing reductions in the amplitude of the oscillations. (B) Schematic (top) and 3D models (bottom) showing two potential Z-shaped binding architectures and crude structures. (C) Probability ratio level predictions for the two Z-shaped structures. (Orange) Corresponding to right schematic in (B) - two proteins that partially wrap the DNA and are positioned out-of-phase. (Blue) Corresponding to left schematic in (B) - two triangle shaped proteins that are positioned out-of-phase. The data shows the reduction in the amplitude of the 6 bp periodicity for the partially wrapped structure. (D) Gel shift data for TetR and TetR-GST as read by an Agilent tape-station. Left panel: gel-like depiction with left, middle, and right lanes corresponding to the ladder, pure DNA, and TetR/TetR-GST bound to DNA. TetR-GST bound to DNA is the thick band below the 1500 bp marker band, and TetR bound to DNA is the band above the DNA band. Alternative depiction of data via absorption plots. Right panel, top: pure DNA absorption. Right panel, bottom: DNA, His-TetR, and GST-TetR absorptions.

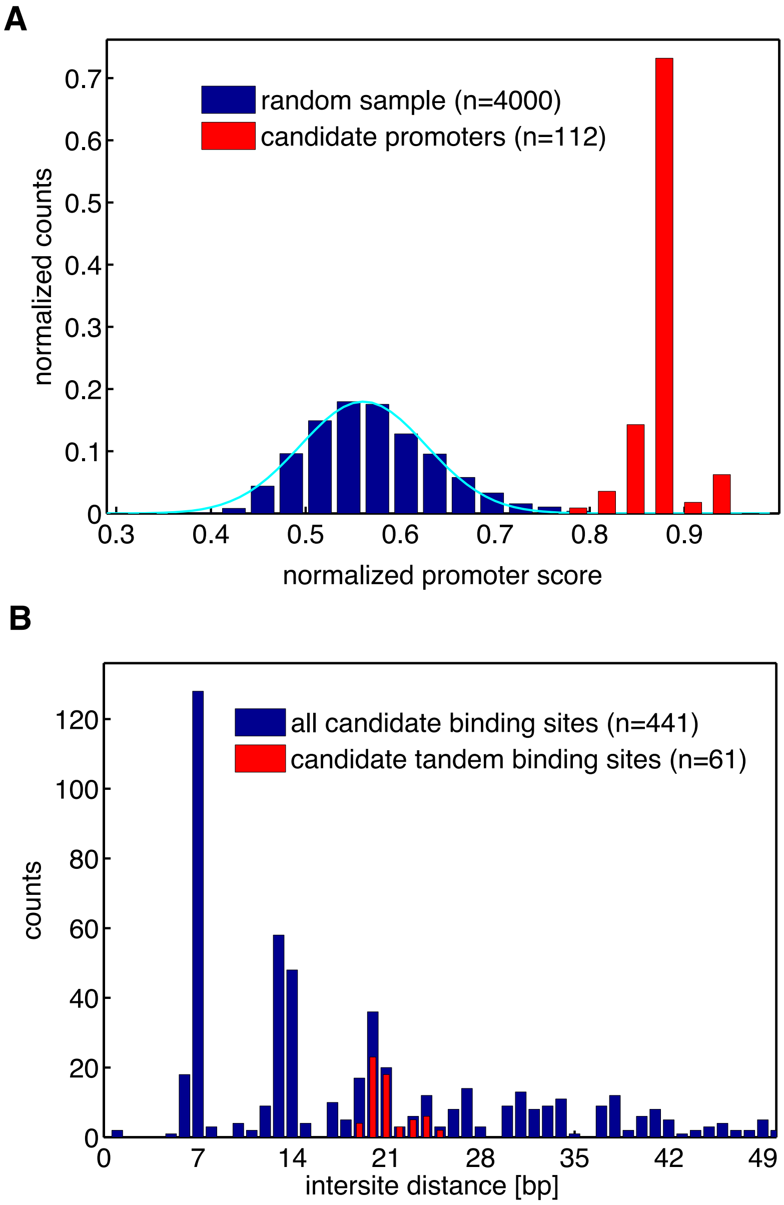

Supplementary Figure 7: Bioinformatic analysis. (A) Distribution of promoter scores for 4000 sequences generated randomly with equal probability for the 4 nucleotides at each position (blue), and for the candidate promoters from the loop sequences (red). All candidate promoters are more than 2σ from the mean of the gaussian fit (cyan) to the random sample. (B) Histogram of inter-site distances for all candidate binding sites (blue) and for the tandem binding sites (red). Range limited to [0, 50] for clarity.

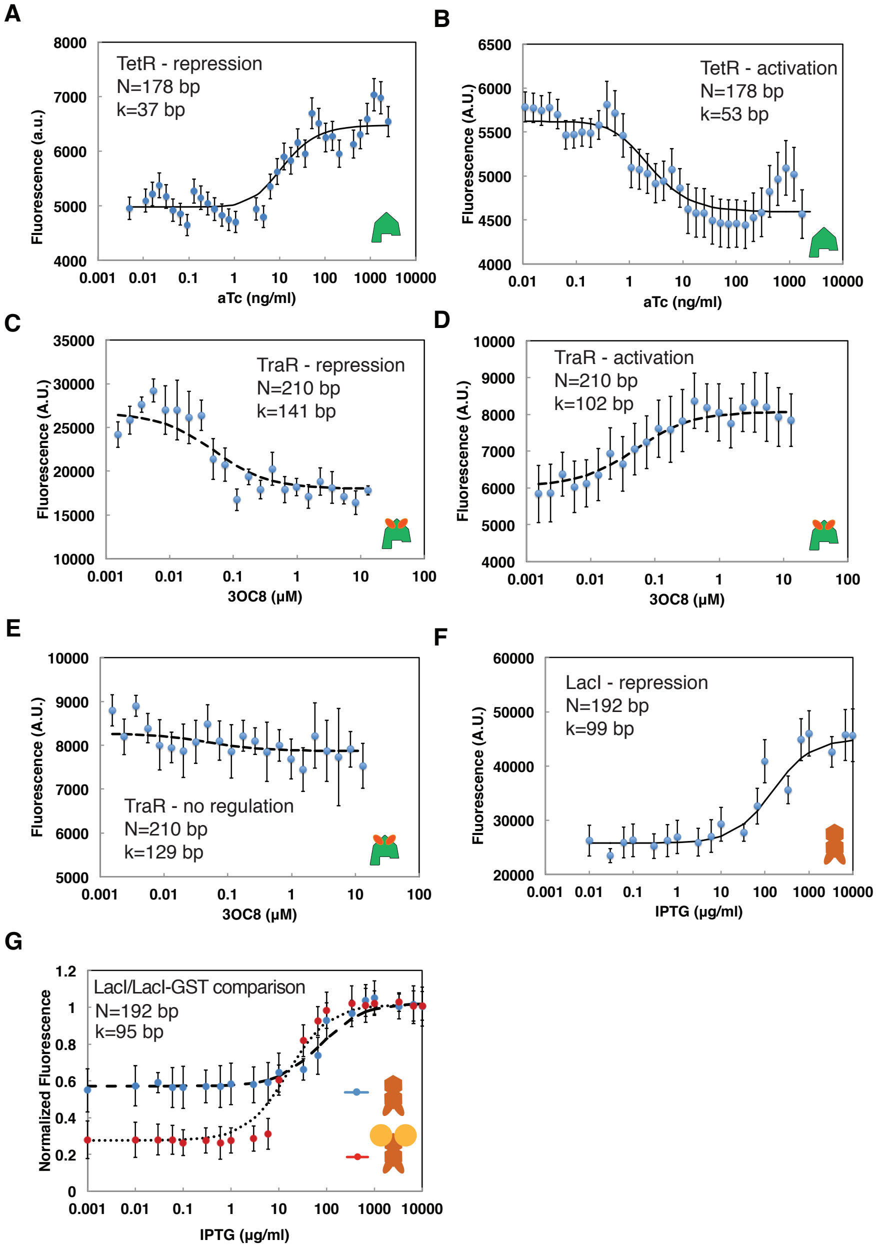

Supplementary Figure 8: Expression level ratio measurements. Six sample data sets showing fluorescence measurements that are subsequently used for the experimental determination of the expression level ratio. The panels plot inducer concentration on the x-axis and raw fluorescence measurements on the y-axis for the synthetic enhancers with looping length N with a single TF binding site displaced k bp away from the driver. All datasets were fit by Hill-1 functions to determine expression level ratio values with error-bars. (A) TetR-repression. (B) TetR-activation. (C) TraR-repression. (D) TraR-activation. (E) TraR - no regulation. (F) LacI-repression. The expression level ratio values determined for these data sets were: (A) $77\% \pm 5\%$, (B) $120\% \pm 5\%$, (C) $67\% \pm 6\%$, (D) $133\% \pm 3\%$, (E) $95\% \pm 5\%$ and (F) $57\% \pm 3\%$. Note that increasing inducer levels has different effects for the different proteins: IPTG and aTc remove LacI and TetR, respectively, from DNA, while 3OC8 enables binding of TraR to DNA. (G) Comparison of dose response functions for LacI and LacI-GST for the single LacI binding site synthetic enhancer set at k=95 bp. LacI-bound synthetic enhancers (blue) and LacI-GST-bound synthetic enhancers (red).

2 Supplementary Tables

nt	1	2	3	4	5	6	7	8	9	10	11	12	13
A	6.6	2.5	9.0	3.3	78.7	55.7	39.3	24.6	3.3	3.3	4.1	78.7	77.9
C	1.6	12.3	2.5	85.2	1.6	5.7	1.6	0.8	7.4	0.8	90.2	2.5	0.8
G	0.8	0.8	82.8	1.6	13.9	4.9	2.5	2.5	1.6	95.1	1.6	6.6	3.3
T	91.0	84.4	5.7	9.8	5.7	33.6	56.6	72.1	87.7	0.8	4.1	12.3	18.0

Supplementary Table 1: Percentage table (depicted pictorially in the inset of Fig. 6) for a single 13-bp LuxO binding site. The consensus site is `T T G C A A/T T/A T T G C A A`.

bacteria	# of NtrC sites	# of ArgR sites	NtrC-Arg4 spacing	Arg43 spacing	Arg32 spacing	Arg21 spacing	Arg1-promoter spacing
E. coli	2	4	110	22	21	31	24
S. typhimurium	2	4	110	21	32	21	22
P. aeruginosa	2	4	45	21	30	21	45

Supplementary Table 2: Spacing between ArgR and NtrC and between the multiple ArgR binding sites for pAstC (*E. coli*, *S. typhimurium*) and pAruC (*P. aeruginosa*).

3 Supplementary Note 1: looping in the context of the wormlike chain (WLC) model

3.1 The discrete WLC model

To evaluate the probability ratio $\hat{R}_n(N,k,s,...)$ theoretically we need to model the looping probabilities $P_{looped,n}(N,k,s,...)$ of the different enhancer configurations. We begin with the simple case of DNA looping without any proteins present. DNA is typically modeled as a discrete semi-flexible chain made of individual links of length l, such that the deviation of one link from its adjacent counterpart depends solely on some elastic bending energy. The chain links do not interact with each other. This class of polymer models is based on the original work of Kratky and Porod [1] and is referred to as the class of wormlike chain models (WLC). This class includes both discrete (i.e., a chain consists of a finite number of links with a certain length) and continuous models.

A chain is described by the locations of its links, and a local coordinate system defined by three orthonormal vectors $\hat{u}, \hat{v}, \hat{t}$ at each link. The vector $\hat{t}$ points along the direction of the chain links. For the continuous WLC these vectors are defined continuously along the chain contour. For the discrete WLC, a chain is defined by its joint locations $\mathbf{r}_i$, along with the local coordinate systems of all links. An example of a discrete WLC of five links, with unit link length is shown in Supp. Fig. 1A.

The elastic energy of the entire chain can be broken into a sum of contributions from individual chain links. The elastic energy of a single ith link in the chain consists of two contributions. The first contribution is the the elastic energy associated with bending link $i \in \{2,...,N\}$ relative to link $i-1$ with angles θ_i, ϕ_i (zenith and azimuthal angles in local spherical coordinates of the previous link). This is the conventional bending energy, which can be written as:

$$\beta E_i^{bend} = \frac{a}{2}\left|\hat{t}_i - \hat{t}_{i-1}\right|^2 = a\left(1-\cos\theta_i\right), \tag{1}$$

assuming azimuthal symmetry, where $\beta = \left(k_b T\right)^{-1}$, T is the temperature, k_b is the Boltzmann factor, and a is the bending constant of the DNA chain. The second contribution accounts for the energy associated with twisting each link in the DNA double helix by Ω_i (twist angle) with respect to the previous link (i.e. rotating $\hat{u}_i$ relative to $\hat{u}_{i-1}$ at an angle of Ω_i). This term is modeled similarly to the term used to describe the twisting energy of a torsion spring [2]:

$$\beta E_i^{twist} = c\left(\Omega_i - \Omega_0\right)^2, \tag{2}$$

where c is the twisting rigidity constant, and where we denote the relaxed twisting angle of the DNA chain (the native twist of ≈ 1.81 radian per nm) by Ω_0. Consequently, the resulting elastic energy is

$$\beta E^{el}\left(\theta_i,\phi_i,\Omega_i\right) = a\left(1-\cos\theta_i\right) + c\left(\Omega_i-\Omega_0\right)^2. \tag{3}$$

Note that all the angles $\theta_i, \phi_i, \Omega_i$ are given in the local coordinate system of the $(i-1)$th link.

We number the links of a chain in the range $1..N$ for a chain of N links. For a specific configuration of the chain we introduce a notation $\{\theta_n, \phi_n, \Omega_n\}$ to denote the set of all the links' angles of the chain, from link 1 to link n. It follows then that the energy of the entire chain consisting of N links is given by

$$E\left(\{\theta_N,\phi_N,\Omega_N\}\right) = \sum_{i=2}^{N} E^{el}\left(\theta_i,\phi_i,\Omega_i\right). \tag{4}$$

The configurational partition function for the model DNA chain consisting of N links immediately follows:

$$Z_N = \int_{-1}^{1} d\cos\theta_2 \int_{0}^{2\pi} d\phi_2 \int_{0}^{2\pi} d\Omega_2 \cdots \int_{-1}^{1} d\cos\theta_N \int_{0}^{2\pi} d\phi_N \int_{0}^{2\pi} d\Omega_N \exp\left[-\beta E\left(\{\theta_N, \phi_N, \Omega_N\}\right)\right]. \quad (5)$$

This model has been extensively studied in the past [3, 4, 2].

3.2 The discrete WLC Monte-Carlo algorithm

The probability of looping for a given choice of parameters $P_{looped,n}(N, k, s, ...)$ can be calculated using a Monte-Carlo algorithm based on the importance sampling method [5] (as described in [6]). The algorithm generates a faithful statistical ensembles consisting of $N_c \approx 10^9$ DNA chains. Any physical observable can then be computed from the generated ensemble by

$$\langle f \rangle = \frac{\sum_{j=1}^{N_c} f\left(\{\theta_N, \phi_N, \Omega_N\}_j\right)}{N_c}. \quad (6)$$

The probability of looping is computed by $P_{looped} = \langle f_{looped} \rangle$ where

$$f_{looped}\left(\{\theta_N, \phi_N, \Omega_N\}_j\right) = \begin{cases} 1 & \text{configuration } j \text{ is looped} \\ 0 & \text{otherwise} \end{cases}. \quad (7)$$

This provides the basis for the self-avoiding wormlike chain model presented in the next section.

4 Supplementary Note 2: looping in the context of the self-avoiding wormlike chain (SAWLC) model

4.1 The discrete SAWLC model

Except for a few notable exceptions [7, 8], the WLC model does not take into account energetic and entropic effects that emerge from the cross-section or "thickness" of the DNA double helix. In order to model the effects of the effective DNA cross-section size, we must take into account an additional contribution to the elastic energy. We engulf each end-point of a link (a joint in the chain) by a "hard-wall" spherical shell of diameter w. An example of such a chain with three links (four joints), and $w = 2.5l$ is shown in Supp. Fig. 1B.

This allows us to model the final contribution to the elastic energy as a set of hard-wall potentials. We denote the end-point of link i as joint i. As previously mentioned, the chain links are numbered $1..N$. Joint 0 is the beginning terminus of the chain. For the simple case in which the chain link length l is larger than the chain diameter ($l \geq w$) and therefore no two neighboring hard-wall spheres overlap, the hard-wall potential energy for the ith chain link is defined as:

$$E_i^{hw}\left(\{\theta_i, \phi_i, \Omega_i\}\right) = \begin{cases} \infty & \text{joint } i \text{ overlaps with one or more joints } 0..(i-1) \\ 0 & \text{otherwise} \end{cases}. \quad (8)$$

This allows us to write an expression for the total elastic energy associated with the chain of spheres as follows:

$$E\left(\{\theta_N,\phi_N,\Omega_N\}\right)=\sum_{i=2}^{N}E^{el}\left(\theta_i,\phi_i,\Omega_i\right)+\sum_{i=1}^{N}E_i^{hw}\left(\{\theta_i,\phi_i,\Omega_i\}\right). \tag{9}$$

We term this model the self-avoiding wormlike chain model (SAWLC). Similarly, Eq. (5) holds for the SAWLC, with the substitution of (9) for (4).

Performing the substitutions (9), (8) into (5) and opening the sums yields:

$$\begin{aligned} Z_N &= \int_{-1}^{1} d\cos\theta_1 \int_0^{2\pi} d\phi_1 \int_0^{2\pi} d\Omega_1 \exp\left[-\beta E^{el}\left(\theta_1,\phi_1,\Omega_1\right)\right]\Theta_i^{hw}\left(\{\theta_1,\phi_1,\Omega_1\}\right)\cdots \\ &\cdots\int_{-1}^{1} d\cos\theta_N \int_0^{2\pi} d\phi_N \int_0^{2\pi} d\Omega_N \exp\left[-\beta E^{el}\left(\theta_N,\phi_N,\Omega_N\right)\right]\Theta_N^{hw}\left(\{\theta_N,\phi_N,\Omega_N\}\right), \end{aligned} \tag{10}$$

where

$$\Theta_i^{hw}\left(\{\theta_i,\phi_i,\Omega_i\}\right)=\begin{cases}0 & \text{joint } i \text{ overlaps one or more joints } 0..\left(i-1\right)\\ 1 & \text{otherwise}\end{cases}, \tag{11}$$

which in turn can be written as:

$$Z_N=\int_{no\ overlap} d\cos\theta_1 d\phi_1 d\Omega_1 \exp\left[-\beta E^{el}\left(\theta_1,\phi_1,\Omega_1\right)\right]\cdots\int_{no\ overlap} d\cos\theta_N d\phi_N d\Omega_N \exp\left[-\beta E^{el}\left(\theta_N,\phi_N,\Omega_N\right)\right]. \tag{12}$$

In the case in which the chain link length l is smaller than the chain diameter ($l < w$), two or more consecutive spheres overlap. This is resolved by introducing Δi such that there can be an interaction between links j, k only if $|j-k| \geq \Delta i \geq \frac{w}{l}$. This changes (11) to:

$$\Theta_i^{hw}\left(\{\theta_i,\phi_i,\Omega_i\}\right)=\begin{cases}0 & \text{joint } i \text{ overlaps one or more joints } 0..\left(i-\Delta i\right)\\ 1 & \text{otherwise}\end{cases}. \tag{13}$$

Recently, we made a significant contribution to the understanding of polymer cyclization or looping by providing a detailed analysis of this phenomenon in the context of the SAWLC [6]. In this work, we first confirmed numerically previous renormalization group predictions [7, 8, 9] and scaling theory [10] models, and subsequently provided new numerical analysis that was applicable to resolving the hyper-bendability effect observed in DNA cyclization experiments for short dsDNA [11, 12]. The algorithm used for the polymer cyclization analysis forms the basis for the algorithm that we describe below.

4.2 The discrete SAWLC Monte-Carlo algorithm

To faithfully sample the configurational space of the SAWLC model we utilized a Monte-Carlo algorithm based on the weighted-biased sampling method [13] to generate statistical ensembles consisting of $N_c \approx 10^7 - 10^9$ DNA chains. In brief, the algorithm generates chains by growing them one link at a time, while systematically checking that the new link does not cross any previous links in the chain. When such a crossing occurs, this fact is taken into account by updating the weighting factor of the chain W. This counter-weight balances the over/under-representation of that chain in the ensemble. For a more detailed description of the method see [6].

Any physical observable f can then be estimated from the generated ensemble using the following formula:

$$\langle f \rangle = \frac{\sum_{j=1}^{N_c} f\left(\{\theta_N, \phi_N, \Omega_N\}_j\right) W\left(\{\theta_N, \phi_N, \Omega_N\}_j\right)}{\sum_{j=1}^{N_c} W\left(\{\theta_N, \phi_N, \Omega_N\}_j\right)}, \quad (14)$$

which includes the probability of looping defined by Eq. 7.

4.3 Protein-DNA interactions and looping in the context of the SAWLC model

4.3.1 Modeling a DNA chain with a bound protein

In order to expand our basic self-avoiding simulation of polymer configurations to model a nucleoprotein structure made of dsDNA and proteins, we first consider some broad geometric characteristics of dsDNA structure. dsDNA is composed of two interwound helical strands, which form a double-helix backbone linked by base-pairs. The backbone exhibits major and minor grooves. The major groove is the wider groove between the two, while the minor groove corresponds to the situation where strands are closer together on the relevant side of the double helix than on the other.

Since most DNA binding proteins bind onto the major groove of the DNA, we must continuously keep track of the location of the major groove along the DNA to properly model protein-DNA binding. To that end, we use one of the vectors defining the local coordinate system of each link: $\hat{u}_i$ (Supp. Fig. 1A). On each link, $\hat{u}_i$ points from the center of the chain's cross-section to the center of the major groove. As a result, we model a protein bound to a binding site of n basepairs, with m being the index of the first basepair of the binding site, by a protrusion from the chain shaped like a sphere with some diameter $w_{protein}$ representative of the protein's volume (Supp. Fig. 1C):

$$\mathbf{r}_{protein} = \mathbf{r}_c + \left(\frac{w + w_{protein}}{2}\right) \hat{u}_c, \quad (15)$$

where we defined the center link of the binding site as

$$c \equiv m + n/2. \quad (16)$$

Note that $\mathbf{r}_c$ is the joint at the end of link c.

4.3.2 Monte-Carlo simulation of a DNA chain with a bound protein

In order to adapt our algorithm to the case of protein-bound DNA, we have to take into account not only the growing chain but also the location of the protrusion as defined by (Eq. 15). During the application of the Monte-Carlo algorithm, upon reaching link $(m + n/2)$ in a specific chain, the simulation attempts to add a hard-wall sphere with diameter $w_{protein}$ at the location $\mathbf{r}_{protein}$. If the sphere overlaps any of the previously generated objects (chain links or other proteins) the chain is discarded, and a new chain is grown from the beginning. The excluded volume of the new sphere is taken into account, when subsequent links are generated. Only completed nucleoprotein chains are properly weighted and counted within the new nucleoprotein configurational distribution.

4.3.3 Modeling bending and stiffening of a thick chain

The excluded volume of the protein bound to a binding site of n basepairs, with m being the index of the first basepair of the binding site, is introduced via a spherical protrusion, as described in section

4.3.1 for the general case. Local DNA bending and stiffening effects induced by the bound protein are simulated via changes applied to the $[m, m+n-1]$ chain links as they are being generated.

Local stiffening of the DNA is simulated by using a different bending constant $a' > a$ (Eq. 1).

Local bending of the DNA by an angle of κ is simulated by introduction of a small rotation of the local coordinates system at each link in the protrusion binding site by κ/n around $\hat{v}_c$ (the unit vector $\hat{v}$ of the center link). This results in a cumulative bending of the chain "around" the bound protein (Supp. Fig. 1D). The energy associated with this additional rotation is zero. To properly generate the bend using our Monte-Carlo approach, the simulation estimates the value of $\hat{v}_c$ of the center link of the binding site for all binding-site links $i \in [m, m+n-1]$ by:

$$\hat{v}_c \approx R_{(c-i)2\pi/P, \hat{t}_i} \hat{v}_i, \tag{17}$$

where P is the helical repeat of the chain ($P \approx 10.5$ for DNA) and $R_{(c-i)2\pi/P, \hat{t}_i}$ is a rotation matrix by an angle $(c-i)\frac{2\pi}{P}$ around $\hat{t}_i$. This approximation is valid for the simulation of DNA since $n/2$ is smaller than the bend and twist persistence lengths of $\sim 50-100$ nm. by at least an order of magnitude (see Simulation Parameters in Materials and Methods). Thus, at these length scales the DNA is expected to be fairly straightand each successive link i is rigidly twisted by $\sim \frac{2\pi}{P}$ around $\hat{t}_i$ relative to link $i-1$.

4.3.4 Looping criterion

After numerically generating the configurational ensemble, the subset of "looped" configurations needs to be properly defined numerically. In the basic cyclization model, looping was defined [6] by having both ends of the polymer within some distance δ. However, unlike the DNA cyclization experiments in which the DNA chain segment simply closes on itself, σ^{54} transcription initiation requires the interaction of two proteins bound to the chain's ends. In particular, the driver interacts with the σ^{54} factor located directly on the DNA beneath the polymerase, thus requiring the driver to access the polymerase complex from the bottom [14]. Therefore, to faithfully model this process via looping, we must add at least two proteins to the ends of the chain - the driver and the polymerase. For simplicity, we neglect the volume of the σ^{54}. We define a looped state as one in which the driver protein interacts with the σ^{54} factor in only a subset of the possible solid angles, corresponding to the σ^{54} acceptance cone and the extent of the GAFTGA loop of NtrC [14]. Thus, the criteria for a looped driver-promoter interaction as they are shown in Supp. Fig. 1E, are as follows:

1. The driver and the σ^{54} factor must be in close proximity, defined by maximal separation of $|\mathbf{r}_{drv} - \mathbf{r}_{\sigma^{54}}| < \frac{w_{drv}}{2} + \varepsilon$.

2. The direction from the DNA to σ^{54} factor is collinear with the the direction from the σ^{54} factor to the driver, within the range $\delta\omega$. I.e. $(\mathbf{r}_{drv} - \mathbf{r}_{\sigma^{54}})$ is collinear with $-\hat{u}_1$ within the range $\delta\omega$. (Conditions 1-2 define a volume $\delta\mathbf{r}$ in which $\mathbf{r}_{drv}$ must be located, illustrated as a green cone in Supp. Fig. 1E).

3. The direction from the DNA to the driver is collinear with the the direction from the driver to the σ^{54} factor, within the range $\delta\omega$. I.e. $(\mathbf{r}_{\sigma^{54}} - \mathbf{r}_{drv})$ is collinear with $\hat{u}_N$ within the range $\delta\omega$ (Illustrated as a light blue cone with its base in $\mathbf{r}_{drv}$ centered around $\hat{u}_N$ in Supp. Fig. 1E).

Using this definition, our algorithm can compute $P_{looped,0}(N)$ for a bacterial enhancer-promoter system of looping length N.

4.3.5 Modeling looping of a thick chain with a bending or stiffening protrusion

Any configuration of bound proteins can be simulated in this way, allowing us to compute $P_{looped,n}(N, k, s, ...)$ in a straightforward fashion. For example, we consider a single TetR transcription factor bound to an enhancer of length 100 bp. The TetR transcription factor is roughly spherical and can be modeled well by a spherical protrusion with diameter of $w_{TetR} = 5.44$ nm, and with a binding site size of $n = 18$ bp, on an enhancer-promoter chain that is $N = 500$ bp long. Consequently, such a protein bound to a binding site spanning links $25 - 42$ is modeled by a sphere located at $\mathbf{r}_{TetR} = \mathbf{r}_{34} + \left(\frac{w+w_{TetR}}{2}\right)\hat{u}_{34}$, according to Eq. (15). Additional stiffening of the DNA by a factor of two is simulated by using $a' = 2a$ during the generation of the links $25 - 42$. Additional bending of the DNA by $\kappa = 10°$ is simulated by 18 instantaneous rotations of the local coordinate system by $\frac{10°}{18}$ during generation of the links $25 - 42$ around the orientation of $\hat{v}_{34}$, approximated at each link $25 \leq i \leq 42$ as described by Eq. (17). This then allows us to generate the properly weighted nucleoprotein ensemble, and compute $P_{looped,n=1}(N = 500, k = 34)$ in a straightforward fashion as defined above.

5 Supplementary Note 3: modeling the experiment

5.1 Thermodynamic modeling of looping-initiated transcription

Our proposed numerical model is capable of calculating the probability of looping for a given experimental setup. The output of the *in vivo* looping experiments, however, is measured indirectly by monitoring the resultant reporter protein fluorescence level when the cells reach steady-state. We need to derive a model that connects the experimental readout to the probability of looping. Since we measure the average fluorescence for a population of cells in our experiments, we only need to provide a prediction for the mean expression levels in our simulations. While a full stochastic model (e.g., [15]) can provide a prediction for the second and higher moments of the distribution, it is has been shown that the stochastic model's expression for the first moment is equivalent to the mean expression level obtained from a thermodynamic equilibrium model, that is constructed using the same assumptions [16].

To model a minimal enhancer, which consists of only the driver binding site and the promoter, we make the following assumptions:

- We assume that the glnAp1 promoter is only active when the concentration of $NRI \sim P$ is vanishingly small (for a justification of this assumption see [17]). When a small amount of $NRI \sim P$ accumulates, the hexameric complex assembles, which simultaneously strongly represses the glnAp1 promoter while activating glnAp2. This means that all transcription in our system is a result of the glnAp2 promoter.

- There is always a bound "poised" polymerase at the promoter awaiting an activation signal. While it was recently shown that the RNAP releases from and rebinds to the promoter frequently [18], it was also previously shown that σ^{54} promoters are mostly occupied by poised polymerases both *in vivo* and *in vitro* [19, 20, 21]. This means that we do not need to include states that lack bound RNAP in our model.

- For the $NRI \sim P$ hexamerization, a cooperative process, the appropriate expression for equilibrium binding is given by:

$$\frac{\left(\frac{[NRI]}{K_{NRI}}\right)^m}{1+\left(\frac{[NRI]}{K_{NRI}}\right)^m}, \tag{18}$$

where $[NRI]$ is the concentration of phosphorylated $NRI \sim P$ dimers, K_{NRI} is the $NRI \sim P$ dissociation constant that incorporates the cooperativity of the binding interaction, and $m > 1$ is some coefficient that signifies the multimerization of $NRI \sim P$ at the two NRI sites. One can expect m to be as high as 6, but it could also be lower since $NRI \sim P$ is a dimer in solution. Hence, we expect $3 < m < 6$ [22]. The subsequent constant production of $NRI \sim P$ in our experiments and the cooperative binding allows us to assume that:

$$\left(\frac{[NRI]}{K_{NRI}}\right)^m \gg 1, \tag{19}$$

which allows us to posit that a driver complex [i.e. $(NRI \sim P)^m$] is always bound at the driver binding sites.

Given these assumption, we only need to model two states: the driver-and-RNAP-occupied enhancer-promoter non-looped state, and the transcriptionally active looped state.

- Finally, we assume that the rates of driver binding, looping, and unlooping are much faster than the subsequent rates involved in transcription. This means that before ATP can be hydrolyzed and an open complex be formed at the promoter, the driver-DNA-σ^{54} complex has sufficient time to equilibrate. This in turn means that the DNA-bound driver complex has sufficient time to explore its conformational space. This assumption is supported by kinetic data recently obtained by [18]. This means that the two relevant states are in thermodynamic equilibrium, and can be modeled accordingly.

Given these assumptions, we begin by writing the rate equation, which describes the kinetics of looping-initiated transcription for a single bacterial enhancer:

$$\frac{d}{dt}[mRNA] = \alpha P_{int}(N) - \beta [mRNA], \tag{20}$$

where $P_{int}(N)$ is the probability of the driver complex bound N links from the polymerase to interact with the polymerase, $[mRNA]$ is the mRNA concentration, α is the rate for transcription per unit volume after the looped structure between the polymerase and driver has formed, and β is an mRNA degradation rate constant. In steady state the fluorescence reporter level is proportional to the number of mRNA transcripts, resulting in:

$$Fl \propto [mRNA] = \frac{\alpha}{\beta} P_{int}(N), \tag{21}$$

where Fl corresponds to reporter protein concentration (or fluorescence level readout). Often the exact values of α, β and the proportion of Fl to $[mRNA]$ are not known.

The probability of the driver and the polymerase to be in close enough proximity to interact is given by:

$$P_{looped}(N) = \frac{\displaystyle\int_{\substack{\text{looped}\\\text{configurations}}} \exp\left[-\beta E_{conf}\right] d^N \xi_i}{\displaystyle\int_{\substack{\text{all}\\\text{configurations}}} \exp\left[-\beta E_{conf}\right] d^N \xi_i}, \tag{22}$$

where ξ_i are the N coordinates that define a conformation, E_{conf} is the energy of a configuration, and $\beta = (k_b T)^{-1}$, where T is the temperature and k_b is the Boltzmann constant. What actually constitutes a "looped configuration" is defined in 4.3.4. In order to write down a simpler expression for the probability of looping, we denote for convenience a general expression for a partial partition function constrained by some condition:

$$\mathcal{Z}_{condition}(N) = \int\limits_{\substack{\text{configurations that}\\ \text{satisfy the condition}}} \exp\left[-\beta E_{conf}\right] d^N \xi_i, \tag{23}$$

where the integral is taken only over those configurations that satisfy the specific condition. This allows us to define the looping probability functions as follows:

$$P_{looped}(N) = \frac{\mathcal{Z}_{looped}(N)}{\mathcal{Z}_{all}(N)} = \frac{\mathcal{Z}_{looped}(N)}{\mathcal{Z}_{looped}(N) + \mathcal{Z}_{non-looped}(N)}. \tag{24}$$

To develop an expression for the interaction probability, we add a configuration-independent driver-polymerase interaction energy denoted by E_{nr} to the energy of a looped configuration E_{conf} . This allows us to define the interaction partition functions as follows:

$$\mathcal{Z}_{interacting}(N) = e^{\beta E_{nr}} \mathcal{Z}_{looped}(N), \tag{25}$$

leading to the following expression for the probability of interactions:

$$P_{int,0}(N) \quad = \quad \frac{e^{\beta E_{nr}} \mathcal{Z}_{looped}(N)}{e^{\beta E_{nr}} \mathcal{Z}_{looped}(N) + \mathcal{Z}_{non-looped}(N)}, \tag{26}$$

where the index 0 corresponds to the number of TF binding sites in the enhancer. Since $\mathcal{Z}_{non-looped}(N) \approx \mathcal{Z}_{all}(N)$, by dividing the numerator and denominator by $\mathcal{Z}_{all}(N)$ we arrive at:

$$P_{int,0}(N) \approx \frac{e^{\beta E_{nr}} P_{looped}(N)}{1 + e^{\beta E_{nr}} P_{looped}(N)} = \frac{\frac{P_{looped}(N)}{K_{nr}}}{1 + \frac{P_{looped}(N)}{K_{nr}}}, \tag{27}$$

where $K_{nr} \equiv \exp(-\beta E_{nr})$ is the dissociation constant of the protein-protein interaction in the looped conformation . For convenience we define the "looping capacity" as:

$$\chi(N) \equiv \frac{P_{looped}(N)}{K_{nr}}, \tag{28}$$

resulting in a compact result for the minimal enhancer interaction probability:

$$P_{int,0}(N) \approx \frac{\chi_0(N)}{1 + \chi_0(N)}. \tag{29}$$

Note, that Amit et al. [17] demonstrated experimentally that the model described above and summarized in Eq. (29) adequately describes the transcriptional kinetics of our $(NRI \sim P)^6 - \sigma^{54}$ system. In addition, Friedman et al. [18] used single molecule kinetics experiments to show that assumptions above are valid via careful measurements of every rate constant in the enhancer-poised promoter complex formation and subsequent transcription initiation.

5.2 Connecting thermodynamic model and experiment

By normalizing the results of one experiment by those of another experiment (see Eq. (21)) we can derive an experimentally measurable expression that both eliminates the need to experimentally determine $\frac{\alpha}{\beta}$ and corresponds to the ratio of the driver-polymerase interaction probabilities $P_{int}^{(i)}$ for the experiments $i = 1, 2$:

$$\frac{Fl^{(1)}}{Fl^{(2)}} = \frac{P_{int}^{(1)}(N)}{P_{int}^{(2)}(N)}. \tag{30}$$

Below, we demonstrate that under certain assumptions that hold for our experimental setup, $\frac{P_{int}^{(1)}(N)}{P_{int}^{(2)}(N)}$ can be replaced by the ratio of the looping probabilities for the two experiments $i = 1, 2$.

Based on the kinetic measurements that were made by [18], we can now estimate the magnitude of $P_{int,0}(N)$. We begin with the rate equations described in their model [18, Fig.7]:

$$\begin{aligned}
\frac{d}{dt}[RP_1] &= 0 = & k_1[DNA][\sigma^{54}] - (k_{-1} + k_2)[RP_1] + k_{-2}[RP_2], \\
\frac{d}{dt}[RP_2] &= 0 = & k_2[RP_1] - (k_{-2} + k_3)[RP_2] + k_5[RP_0], \\
\frac{d}{dt}[RP_0] &= 0 = & k_3[RP_2] - (k_5 + k_4)[RP_0], \\
\frac{d}{dt}[\sigma^{54}] &= 0 = & -k_1[DNA][\sigma^{54}] + k_{-1}[RP_1] + k_4[RP_0],
\end{aligned} \tag{31}$$

where $[\sigma^{54}]$ is the concentration of available σ^{54}, RP_1 and RP_2 are two types of closed complexes in which DNA remains base-paired, and RP_0 is the open complex in the looped configuration (interacting state). Solving for the fraction of DNA in interacting state, and plugging in the values found by [18], we can extract the probability for finding the driver-polymerase in a bound-looped state RP_0 as follows:

$$P_{int,0} = \frac{[RP_0]}{[RP_0] + [RP_1] + [RP_2]} = \frac{k_2 k_3}{k_2(k_3 + k_4 + k_5) + k_3 k_4 + k_{-2}(k_4 + k_5)} = 0.0101. \tag{32}$$

This implies (see Eq. (29)) that $P_{int,0}(N) \approx \frac{\chi_0(N)}{1+\chi_0(N)} \ll 1$, and therefore

$$P_{int,0}(N) \approx \frac{\chi_0(N)}{1 + \chi_0(N)} \approx \chi_0(N). \tag{33}$$

Using Eqs. (30), (33) and (28), we obtain

$$\frac{Fl^{(1)}}{Fl^{(2)}} = \frac{P_{int}^{(1)}(N)}{P_{int}^{(2)}(N)} \approx \frac{P_{looped}^{(1)}(N)}{P_{looped}^{(2)}(N)} \tag{34}$$

for the experiments i=1,2. This expression enables direct comparison of our experimental measurements to the numerical simulations. Finally, we note that since [18] used DNA segments of comparable lengths to the ones modeled by us, we will assume that Eq. (33) and the assumption

$$\chi_n(N) \ll 1 \tag{35}$$

hold for all looping capacities (all n) considered in this work.

5.3 Enhancer with transcription factor (TF) binding site

When the enhancer contains one TF binding site ($n = 1$) we can write the probability of interaction in a similar fashion to Eq. (27):

$$P_{int,1}(N,k,[TF]) = \frac{e^{\beta E_{nr}} \mathcal{Z}_{\substack{\text{looped}\\ \text{no TF}}}(N) + \frac{[TF]}{K_{TD}} e^{\beta E_{nr}} \mathcal{Z}_{\substack{\text{looped}\\ \text{bound TF}}}(N,k)}{e^{\beta E_{nr}} \mathcal{Z}_{\substack{\text{looped}\\ \text{no TF}}}(N) + \frac{[TF]}{K_{TD}} e^{\beta E_{nr}} \mathcal{Z}_{\substack{\text{looped}\\ \text{bound TF}}}(N,k) + \mathcal{Z}_{\substack{\text{non-looped}\\ \text{no TF}}}(N) + \frac{[TF]}{K_{TD}} \mathcal{Z}_{\substack{\text{non-looped}\\ \text{bound TF}}}(N,k)}, \tag{36}$$

where $k < N$ is the number of base pairs between the center of the driver binding site and the center of the TF binding site and K_{TD} is the binding constant of the TF to its binding site. Again, by dividing the numerator and the denominator by $\mathcal{Z}_{all}(N)$:

$$P_{int,1}(N,k,[TF]) \approx \frac{\chi_0(N) + \frac{[TF]}{K_{TD}} \chi_1(N,k)}{1 + \chi_0(N) + \frac{[TF]}{K_{TD}} (1 + \chi_1(N,k))}, \tag{37}$$

where χ_0 and χ_1 denote the looping capacities for an enhancer without a bound TF (equal to the looping capacity of the minimal enhancer) and the looping capacity for an enhancer with a TF bound to it, respectively. To quantify the effect of TF binding on transcription, we divide the reporter concentration with available TF by the reporter concentration obtained without TF . Following Eqs. (30), (29) and (37) we obtain:

$$r_1(N,k,[TF]) \equiv \frac{Fl_1(N,k,[TF])}{Fl_0(N)} = \frac{P_{int,1}(N,k,[TF])}{P_{int,0}(N)} \approx \frac{1 + \frac{[TF]}{K_{TD}} \frac{\chi_1(N,k)}{\chi_0(N)}}{1 + \frac{[TF]}{K_{TD}} \left(\frac{1+\chi_1(N,k)}{1+\chi_0(N)} \right)}. \tag{38}$$

By performing the experiment with saturating concentrations of the TF we can quantify the maximal regulatory effect:

$$R_1(N,k) \equiv \lim_{[TF]\to\infty} r_1(N,k,[TF]) \approx \frac{\frac{\chi_1(N,k)}{\chi_0(N)}}{\frac{1+\chi_1(N,k)}{1+\chi_0(N)}}. \tag{39}$$

Further simplifying with Eqs. (33) and (35) yields:

$$R_1(N,k) \approx \frac{\chi_1(N,k)}{\chi_0(N)} \approx \frac{P_{looped,1}(N,k)}{P_{looped,0}(N)} \equiv \hat{R}_1(N,k), \tag{40}$$

where $P_{looped,n}$ is the probability of looping with n TFs bound. Eq. (40) thus provides the connection between the experimental expression level ratio of saturating and zero TF concentrations $R_1(N,k)$ and the theoretical looping probability ratio $\hat{R}_1(N,k)$. The latter can be obtained directly from the looping probabilities $P_{looped,1}(N,k)$ and $P_{looped,0}(N)$, which are computed using the numerical Monte-Carlo simulation.

5.4 Enhancer with two TF binding sites

In this case ($n = 2$), we define an additional parameter s to denote the number of base pairs between the center of the first TF binding site and the center of the second TF binding site. In a similar fashion to the one binding site case, we obtain:

$$P_{int,2}\left(N,k,s,[TF]\right) \approx \frac{\chi_0\left(N\right) + \frac{[TF]}{K_{TD}}\chi_1\left(N,k\right) + \frac{[TF]}{K_{TD}}\chi_1\left(N,k+s\right) + \left(\frac{[TF]}{K_{TD}}\right)^2 \chi_2\left(N,k,s\right)}{1+\chi_0\left(N\right) + \frac{[TF]}{K_{TD}}\left(1+\chi_1\left(N,k\right)+\chi_1\left(N,k+s\right)\right) + \left(\frac{[TF]}{K_{TD}}\right)^2\left(1+\chi_2\left(N,k,s\right)\right)}, \tag{41}$$

$$\begin{aligned} r_2\left(N,k,s,[TF]\right) &\equiv \frac{Fl_2\left(N,k,s,[TF]\right)}{Fl_0\left(N\right)} = \frac{P_{int,2}\left(N,k,s,[TF]\right)}{P_{int,0}\left(N\right)} \\ &\approx \frac{1+\frac{[TF]}{K_{TD}}\frac{\chi_1(N,k)+\chi_1(N,k+s)}{\chi_0(N)} + \left(\frac{[TF]}{K_{TD}}\right)^2\frac{\chi_2(N,k,s)}{\chi_0(N)}}{1+\frac{[TF]}{K_{TD}}\left(\frac{1+\chi_1(N,k)+\chi_1(N,k+s)}{1+\chi_0(N)}\right) + \left(\frac{[TF]}{K_{TD}}\right)^2\left(\frac{1+\chi_2(N,k,s)}{1+\chi_0(N)}\right)}, \end{aligned} \tag{42}$$

$$R_2\left(N,k,s\right) \equiv \lim_{[TF]\to\infty} r_2\left(N,k,s,[TF]\right) \approx \frac{\frac{\chi_2(N,k,s)}{\chi_0(N)}}{\frac{1+\chi_2(N,k,s)}{1+\chi_0(N)}} \approx \frac{P_{looped,2}\left(N,k,s\right)}{P_{looped,0}\left(N\right)} \equiv \hat{R}_2\left(N,k,s\right). \tag{43}$$

Here $\chi_2(N,k,s)$ is the looping capacity for an enhancer of length N with two TFs bound at k and $(k+s)$. As with Eq. (40), Eq. (43) provides the connection between the experimental observations and the looping probabilities calculated by our simulations.

6 Supplementary Note 4: supporting data

6.1 Supplementary model data for Figure 1: A geometric model for a maximal regulatory effect at the $k = N/2$ position

In order to provide an explanation for the non-monotonic increase of the regulatory effect as k approaches $N/2$ observed both for our model (Fig. 1) and experiments with TraR (Fig. 2B, 3C), LacI-GST (Fig. 4A), and TetR (Figure 2D), we chose to analyze the most-likely looped configuration ζ_0 predicted by our model. For simplicity, we neglected the volume of the chain, i.e., we used the WLC model. Discrete minimal-energy looped configurations were found for $N = 10, 20, 50$ as the numerical solutions that minimize the total configuration energy of Eq. (4), with boundary conditions that satisfy the ideal looping criterion, namely that the chain ends coincide. For comparison, solutions were approximated to continuous curves $\tilde{\mathbf{r}}_N(uNl)$ with $u \in [0,1]$ and then renormalized to unit length $\mathbf{r}_N\left(u\right) = \tilde{\mathbf{r}}_N\left(uNl\right)/Nl$. The curves for the different N values were found to collapse onto a single curve $\mathbf{r}_{\zeta_0}\left(u\right)$, indicating that the minimal energy loop ζ_0 is independent of N.

Supp. Fig. 2A shows ζ_0 on a unit length curve, parametrized by $u \in [0,1]$, in the loop plane. Note that the minimal energy loop for the 3D problem is in fact two-dimensional. The figure shows that the minimal-energy (or most-likely-to-occur) loop is not a circle as might conventionally be thought, but rather shaped like a teardrop. The implications of this geometry can be seen in Supp. Fig. 2B, where we plot the numerically-calculated squared curvature $\left|\frac{dt_{\zeta_0}}{du}\right|^2$ of the minimal energy loop ζ_0 as a function of the normalized length u. The figure shows that the region of highest curvature is at $u = \frac{1}{2}$, precisely half-way along the contour, whereas the curvature near both ends is very small. This indicates that it is energetically preferential for loops to be more curved near the center of the loop than close to the ends, and loops whose shapes are the same as or closely resemble the minimal loop ζ_0 will be most probable.

We now calculate the curvature-dependence of the energy of link j, for a particular configuration ζ. This is certainly valid in the elastic regime, i.e. for chains whose length is $N \lesssim b$. From Eq. (1):

$$E_j^{\zeta} = \frac{a}{2}\left|\hat{t}_{j+1} - \hat{t}_j\right|_{\zeta}^{2} = a\left(1 - \cos\theta_{j+1}\right). \tag{44}$$

In the limit of $N \gg 1$, the discrete curve along the points $\{\mathbf{r}_0, ..., \mathbf{r}_N\}$ can be approximated with a continuous curve $\tilde{\mathbf{r}}(s)$ parametrized by $s \in [0, Nl]$, which yields:

$$\frac{\left|\hat{t}_{j+1} - \hat{t}_j\right|^2}{l^2} \approx \left|\frac{d\tilde{\mathbf{t}}(s)}{ds}\right|_{s=jl}^{2}. \tag{45}$$

After renormalizing $\tilde{\mathbf{r}}(s)$ to a unit length curve $\mathbf{r}_{\zeta}(u) = \tilde{\mathbf{r}}(s)/Nl$ parametrized by $u \in [0, 1]$, we obtain:

$$\left|\hat{t}_{j+1} - \hat{t}_j\right|^2 \approx \frac{1}{N^2}\left|\frac{d\mathbf{t}_{\zeta}(u)}{du}\right|_{u=j/N}^{2} = \frac{1}{N^2}\kappa_{\zeta}^2(u), \tag{46}$$

where $\kappa_{\zeta}(u) = \left|\frac{d\mathbf{t}_{\zeta}(u)}{du}\right|$ is the curvature, which depends only on the geometric shape of the specific configuration ζ. Thus the energy contribution at the jth link is approximated by:

$$E_j^{\zeta} \approx \frac{a}{2}\frac{1}{N^2}\kappa_{\zeta}^2\left(\frac{j}{N}\right). \tag{47}$$

We now ask where to expect the maximal regulatory effect for a bound TF, based on the shape of ζ_0 and on Eq. (47). Since the energy depends on the squared curvature $\kappa_{\zeta}^2\left(\frac{j}{N}\right)$ and the curvature was found numerically to be maximal at $j = N/2$, we expect a TF at the $N/2$ position to produce the greatest change in energy, and thus to produce the strongest regulatory effect. We consider the separate effects of the bound TF. Stiffening (increasing a) hinders curving in all directions (including the loop-forming direction), which increases the energy of the ζ_0 -like configurations and results in a down-regulatory effect. Bending either assists curving in the loop-forming direction (reduces energy) or hinders curving (increasing energy), depending on the direction of the bending, resulting in an up- or down-regulatory effect, respectively. A protrusion hinders curving in the loop-forming direction if it is positioned "within the loop" since curvature is limited by the presence of the protrusion. Similarly, a protrusion assists curving in the loop-forming direction if it is positioned "outside the loop" by limiting the curvature in the non-loop-forming direction.

6.2 Supplementary model data for Figure 3: a thick chain with two protrusions

In order to model the regulatory effect of two binding sites there is an additional control parameter added into the problem: the inter-site spacing, s. This implies that instead of the 2D heat map for the possible regulatory responses (Fig. 1C), we need to explore a 3D map of potential responses (N, s, k). In Supp. Fig. 3A-B we show two 2D cross-sections of the 3D heat maps: N vs. s at a constant value of k=42 links, and N vs. k at a constant value of s=21 links. In the 2D cross-sections, we notice the following: first, protrusions that are positioned "in phase" to the driver and themselves (Supp. Fig. 3A - blue patches) generate a strong repression signature as expected. Second, when the protrusions are positioned "out-of-phase" with respect to each other (e.g. Supp. Fig. 3A, s=26 links - green-yellow patches), only one protrusion is "in-phase" with the driver, while the other counteracts its effect. As a result, the number of looping configurations excluded by the "in-phase" protrusion is roughly equal to the number excluded in the case of a single protrusion, but the total number of available configurations is reduced by the "out-of-phase" protrusion, leading to a reduced regulatory effect. A closer examination of the in-phase tandems reveals (Supp. Fig. 3B) a similar heat map to

the 2D heat map presented in Fig. 1C, but with a significantly greater amplitude of oscillations between the down-regulation minima and up-regulation maxima.

6.3 Supplementary experimental data for Figure 3: tandem TraR synthetic enhancers with varying looping length and constant s

We carried out additional measurements for tandem TraR synthetic enhancers, keeping the binding site spacing (s) and distance to the driver (k) constant while varying the looping length (N), as follows: in-phase - s=23 bp spacing and k=54 bp, and out-of-phase - $s = 28$ bp and $k = 54$ bp. The expression level ratio data and probability ratio model predictions are plotted in Supp. Fig. 4A and 4C, respectively, with a schematic of the two synthetic enhancer designs shown in Supp. Fig. 4B. Supp. Fig. 4A shows that both experimental data sets exhibit similar trends. The in-phase synthetic enhancers ($s = 23$ bp, blue dots) exhibit strong repression at small looping lengths (~30%), and for larger looping lengths a fluctuating regulatory response that converges on a repression value of 80% independent of the looping length. Despite the strong fluctuations, we also seem to be observing a faint periodic signature characterized by 4 cycles with a periodicity of ~11 bp centered on peaks in expression level ratio for looping lengths $N = 148, 160, 171, 181, 192$. For the out-of-phase data set ($s = 28$ bp, red squares), the expression level ratio shows a similar trend, which is characterized by a weaker over-all regulatory response. The repression at short looping lengths is significantly weaker (70%) as compared with the in-phase case (30%), the convergence to a constant repression value seems to occur at a shorter looping length ($N = 120$ bp vs $N = 140$ bp), and the overall long looping length regulatory response seems to hover around the barely detectible expression level ratio value of 95%. Thus, the out-of-phase arrangement of the TraR binding site produces an expression level ratio behavior which is weaker than the in-phase arrangement, and overall is distinguishable from the in-phase expression level ratio response.

In order to see if our model captures this behavior, we compared two constant k cross-sections across heat maps that were computed with $s = 21$ bp (Supp. Fig. 3B, dashed line) and $s = 26$ bp (Fig. 5A). Here, the model once again captures the experimental trends quantitatively. As in the experiment, the thick chain with "out-of-phase" protrusions produces a significantly different probability ratio level response as compared with the in-phase arrangement. The model shows (Supp. Fig. 5C) that the out-of-phase probability ratio level ($s = 26$ bp, red) produces a weaker response at lower looping lengths (60% vs ~5-10%), converges to a steady state repression level at a lower looping length (150 bp vs 230 bp), and settles on an average weaker repression level (90% vs 80%) than for the in-phase arrangement ($s = 21$ bp, blue). Interestingly, the in-phase variable N model cross-section shows a sharp oscillatory function for all looping lengths, while the out-of-phase and the single binding site variable N cross-sections (Fig. 1E) do not exhibit a detectible 11 bp periodic signal across all looping lengths.

6.4 Supplementary model data for Figure 4

6.4.1 Combined excluded volume and stiffening

In order to simulate the effects of stiffening using the SAWLC model, we modified the simulation by allowing the protrusion to also stiffen the thick chain locally by increasing the bending constant from a to $2a$ at the links attributed to a bound protein . Supp. Fig. 5A shows the effect of a purely-stiffening region on a thick chain in a 2D heat map, while Supp. Fig. 5B shows the effect of a single protrusion that also stiffens the chain (see schematic at the top). Both heat-maps show that a bias towards the quenching domain forms and persists for N >300 bp. The addition of the excluded volume effect (Supp. Fig. 5B) generates a similar checker-board pattern to the ones shown

in Figure 1, but this time with oscillations (see also Fig. 4C) between quenching states only in the region affected by the stiffening of the chain.

6.4.2 Combined excluded volume and bending

Likewise, in order to simulate the effects of bending using the SAWLC model, we modified the simulation by allowing the protrusion to also bend the thick chain locally by introducing small cumulative bends at the links attributed to a bound protein (see section 4.3.3). Supp. Fig. 5C shows the effect of a purely-bending region by an angle of 10º on a thick chain in a 2D heat map, while Supp. Fig. 5D shows the effect of a single protrusion that also bends the chain by an angle of 10º over 16 bp. In Supp. Fig. 5C the data show a similar checker-board pattern to the ones shown in Figure 1, however the magnitude of the regulatory effect is significantly higher than the one produced by excluded volume effect alone. In addition, the locations of the maxima and minima are opposite to those in Figure 1, due to the geometry of bending. A protrusion bound on the same side of the polymer as the activator impedes the chain's ability to bend in the direction of the protrusion, thus making formation of a loop less probable. In contrast, a bending protrusion facilitates the chain to bend in the direction of the protrusion (Supp. Fig. 1D). As a result, bending and excluded-volume have opposite effects. Addition of the protrusion excluded-volume to the bending effect (Supp. Fig. 5D) diminishes the magnitude of the regulatory effect, due to the competition between the two opposing effects. The locations of the minima and maxima of the combined effect depend on the size of the protrusion. For a small-sized protrusion, the bending effect dominates, and the locations of the minima and maxima correspond to Supp. Fig. 5C-inset. As the size of the protrusion is increased, the locations of the minima and maxima are shifted towards Figure 1, until the original order of Figure 1 is restored with the use of a large enough protrusion (Supp. Fig. 5D-inset).

6.5 Supplementary model and experimental data for Figure 5

6.5.1 Experimental results and modeling: LacI-GST out-of-phase tandems

We carried out measurements of the regulatory effect of synthetic enhancers bound by tandem of LacI-GST proteins in out-of-phase orientation (s=38 bp). The expression level ratio data is shown in Supp. Fig. 6A, together with the expression level data obtained for the synthetic enhancers bound by out-of-phase native LacI proteins previously shown in Fig. 5C. As for the case of the synthetic enhancers with tandem vs. single binding sites, the effect of LacI-GST is to increase the overall repression effect due to its larger size. This bias of the regulatory function towards a lower repression value is observed for all synthetic enhancers, as was shown in Fig. 5. Interestingly, however, while the overall trend of repression as a function of displacement (k) is conserved for both the native and fusion protein, the LacI-GST tandem does not seem to exhibit the expected 6 bp oscillations observed with LacI. Rather, the regulatory response seems to be constant with only the longer range looping length dependencies observed.

We reasoned that the loss of detectible oscillations could be due to the structure of LacI-GST, which presumably partially wraps the DNA. To crudely model this structure we simulated the thick chain with two out-of-phase protrusions made of three tangentially-attached spheres of the same size (radii of 2.72 nm), generating a partially wrapping structure when bound to DNA (Supp. Fig. 6B). Specifically, we compared two types of protrusions: a "triangle"-shaped protrusion (Supp. Fig. 6B-left) with additional spheres at 45° relative to $\hat{u}_i$, and partially wrapping protrusion with additional spheres at 45° relative to $-\hat{u}_i$ (Supp. 6B-right). We reasoned that as an out-of-phase tandem, the two aspherical protrusions should form partial arcs around the thick chain, whose excluded volume sensitivity to orientation should be diminished as compared with a spherically symmetric protrusion.

In Supp. Fig 6C, we show the model prediction for both types of protrusions when positioned in out-of-phase tandems on a thick chain. The model shows that the amplitude for the partially wrapping protrusion is significantly reduced as compared with the “triangle” shaped protrusion, which exhibits a more pronounced 6 bp periodicity. Thus, we can conclude that the partially-wrapping protrusion generates a more consistent probability ratio prediction with the expression level ratio measurements, and is more likely to reflect the underlying structure of LacI-GST.

6.5.2 Experimental results: gel shift assay for TetR

In order to test the dumbbell binding structure of TetR(B) to dsDNA, we carried out gel-shift experiments with purified His-tagged TetR and TetR-GST. We reasoned that purified TetR that binds to DNA as a dimer of dimers should do so not only as a homodimer of dimers (TetR-TetR - TetR-TetR), but as a heterodimer of dimers (TetR-TetR - TetR-GST-TetR-GST) as well. We assumed that both TetR and TetR-GST would already be in dimer form before mixing. Thus, a 200 bp piece of DNA containing the TetR binding site and the suspected secondary cryptic site should exhibit three discrete shifted gel bands for TetR-TetR, TetR-TetR-GST, and TetR-GST - TetR-GST complexes. In Supp. Fig. 6D we show the results for the gel shift experiment carried out with a mix of His-TetR-GST and His-TetR after properly calibrating for a 1:1 binding ratio for the two TetR protein species (data not included). The data show only two shifted bands with respect to the non-bound DNA (242 bp): the shifted band that appears for purified TetR (440 bp - middle band, presumably TetR dimer), and the band that appears for purified TetR-GST (850 bp - top band, presumably TetR-GST dimer). Thus, while we have strong evidence for the formation of a dimer-of-dimer structure *in vivo*, the gel shift experiment at the very least seems to rule out the formation of a heterodimer-of-dimer structure *in vitro*, and does not provide additional support for the structural interpretation of our *in vivo* data.

6.6 Supplementary bioinformatics data for Figure 6: pAstC/pAruC promoters

To provide further support to the observations described in Fig. 6, we carried out a comparative study on the annotated pAstC/AruC promoter in *E. coli*, *S. typhimurium*, and *P. aeruginosa.* In all three cases a σ^{54} promoter is driven by NtrC or its *P. aeruginosa* homologue CbrB to generate expression of the *ast*/*aru* genes. In Supp. Table 2 we depict the structure of these bacterial enhancers as a function of their location on the genomes, and values of control parameters. By examining this table it is clear that once again structure is remarkably conserved. The enhancers for *E. coli* and *S. typhimurium* are almost precisely conserved, in terms of looping length and positioning of the ArgR sites. The only difference seems to be the shifting of the site by roughly a helical periodicity upstream. It is important to note that ArgR binds a tandem of sites to form a dimer-of-trimers, which is capable of bending DNA by roughly 70ř. This implies that the four sites really should be viewed as a tandem of binding sites . For *S. typhimurium* and *P. aeruginosa* this architecture is particularly conserved, with each tandem separated by approximately three helical periodicities. In *E. coli* this symmetry is broken by having three half-sites closely positioned, and an additional half-site separated from the *arg432* cassette. The significance of this is unclear. However, the conservation of *argR* arrangement between *P. aeruginosa* and *S. typhimurium*, and the overall conservation of enhancer size and binding site locations between *S. typhimurium* and *E. coli* (except for the shift in the binding site Arg2) are highly consistent with the previous observations for the *qrr* genes.

7 Supplementary Methods

7.1 Protein expression and purification

His-TetR and His-GST-TetR were sub-cloned into the pet28a vector for over-expression and transfected into *E. coli* BL21-Gold (DE3) cells. Following IPTG induction of expression, lysis of cells by homogenization and clearing of the supernatant by centrifugation, the lysate was loaded on a Ni^{++}-Sepharose 6 Fast Flow resin (GE Healthcare). The matrix was washed, eluted with A300 buffer [20 mM Tris (pH 7.5), 10% glycerol, 300 mM KCl, 0.1 mM EDTA] containing 0.2 M imidazole. The eluted fractions were analyzed by SDS–PAGE and those containing His-TetR or His-GST-TetR were pooled and dialyzed against A100 buffer [20 mM Tris (pH 7.5), 10% glycerol, 100 mM KCl, 0.1 mM EDTA and 2 mM DTT]. Purified proteins were verified by coomassie staining.

7.2 Electrophoretic mobility shift assays

Electrophoretic mobility shift assay (EMSA) reactions were performed in binding buffer (10 mM Tris–HCl pH 7.4, 50 mM NaCl, 0.5 mM EDTA pH 8.0, 1 mM $MgCl_2$, 2 mM DTT and 4% glycerol). Reactions containing either His-TetR or His-GST-TetR proteins and DNA cassette with a single TetR binding site were performed at room temperature over the course of 20 min. As a control, we used a DNA cassette with a single TraR binding site, and aTc was added to the sample to a final concentration of 0.1 mM. Samples were analyzed using an Agilent 2200 TapeStation and High Sensitivity D1K ScreenTape or by loading samples onto 8% non-denaturing polyacrylamide gel at 80 V in TBM buffer containing 0.09 M Tris, 0.09 M boric acid and 5 mM $MgCl_2$. DNA run on PAGE was detected by ethidium bromide staining.

7.3 The *Vibrio* genus *qrr* genes

The *Vibrio* genus of bacteria contains many sequenced genomes of marine pathogens. These bacteria have been used as model systems extensively, particularly for the characterization of quorum sensing systems. Quorum regulating RNA (Qrr) are part of the complex regulatory pathway of quorum sensing in *Vibrio.* The *qrr* genes are located on both chromosomes: *qrr1* is on the large chromosome, while *qrr2-5* are located on the smaller chromosome. Quite a few studies have been carried out on these genes [23, 24]. All sequenced *Vibrio* species carry the *qrr1* gene on the larger chromosome immediately adjacent to the sequence encoding LuxO. However, only a subset of the species carry either an additional three or four *qrr* genes on the smaller chromosome [25]. Not much is known about the promoters regulating the transcription of these genes, except for one notable study carried out on the the *V. cholerae qrr4* promoter [24]. Given this partial understanding of the promoters, this system can be used as a case study to test the INDEL prediction.

7.3.1 Annotating the *qrr* σ^{54} promoters

Given that it was shown that the *qrr2-4* in *V. cholerae* are activated by the NtrC-like phosphorylated version of LuxO (LuxO-P) [23, 24], we hypothesized that other genes in other *Vibrio* species are also activated by LuxO from σ^{54} promoters. To test this, we first conducted a BLASTN [26] search of nucleotide databases using a conserved sequence of 32 nucleotides common to all known *qrr* genes (query: GGGTCACCTAKCCAACTGACGTTGTTAGTGAA) [23, 25], and discarded all hits above an e-value threshold of 10^{-5}. We downloaded the full sequences of all remaining hits (169 hits from 86 unique sequences) and extracted the *qrr* gene and 500 bp upstream of the gene for each hit. We then discarded all sequences shorter than 105 bp and all identical 500 bp sequences (leaving one of

each). The remaining set contained 114 hit sequences with unique 500 bp sequences upstream of the hit start position (see Supplementary Data 2).

After identifying the relevant genes, we extracted the sequence that was located immediately upstream of the *qrr* genes in order to annotate the putative σ^{54} promoters. σ^{54} promoters are located within 30 bp of the transcriptional start site (TSS), and exhibit strongly conserved sequences, especially around the -24/-12 positions. We based our search on a consensus sequence derived from [27], a large-scale analysis of 186 -24/-12 promoter elements from 47 different bacterial species. Interestingly, unlike σ^{70} promoters, the consensus sequence for the σ^{54} promoter is highly conserved across many bacterial species, which makes annotating these promoters a straightforward exercise. Consequently, to narrow our search, we examined the first 50 bp upstream of the BLASTN hit start position. Each position in the the 50 bp window was scored with the likelihood of the promoter to start at its site based on 16-bp σ^{54} consensus matrix taken from [27], where we divided the non-consensus weight equally among the non-consensus nucleotides at each position. Scores were then normalized to a 0-1 range by subtracting the minimum possible score and dividing by the maximum possible score. Only promoters with normalized scores greater than 0.744 were accepted, based on the distribution of scores for 4000 randomly-sampled sequences (see Supp. Fig. 7A). This allowed the identification of a single putative σ^{54} promoter for all 114 hits. We continued analysis for the 112 of these sequences that had unique 500 bp windows upstream of the promoter.

7.3.2 Extracting the LuxO binding sites

After identifying the promoters, we next proceeded to identify the LuxO-driver binding sites. Since LuxO is considered to be homologous to members of the NtrC family of response regulators [28], we hypothesized that as for the *V. cholerae* case, LuxO should drive expression from a tandem of binding sites that are spaced approximately two helical repeats (22 bp) apart. Finally, as with other NtrC-family members, we expected the tandems to be located several tens to a couple of hundred basepairs upstream of the putative σ^{54} promoter.

To find the LuxO binding site tandems, we searched the 500 bp upstream of the respective putative σ^{54} promoters. These upstream sequences were first scanned for 13 bp sequences that were candidate LuxO binding sites based on the only annotated LuxO sites in *V. cholerae*: TTGCATTTTGCAA and TTGCAATTTGCAA [24]. We chose a threshold of 9 of the 13 bases that had to match one of the two *V. cholerae* LuxO sites. We then computed the separation between the centers of any two candidate sites in the same upstream region. The center-to-center separation distribution is plotted in Supp. Fig. 7B. In the plot we observe three distinct peaks: at 7 bp, 13 bp, and 21 bp. The 7 bp peak corresponds overlapping binding sites, which are highly probable due to the fact that each binding site is almost an exact double repeat of TTGCAA, and thus this peak can be discounted. The peak at 13 bp is likely also an artifact due to the repetitive nature of the binding sites, with the first of the two sites overlapping the first TTGCAA of the real binding site, and the second binding site overlapping the second TTGCAA. This peak may additionally corresponds to adjacent binding sites without separation, but these are not of interest due to the low likelihood of physical binding of proteins next to one another. Finally, The peak at 19-25 bp corresponds to the desired tandem separation, and these pairs of sites were chosen to be the putative LuxO binding site tandems. Some of the upstream sequences had more than one potential tandem. In these cases, we chose the tandem that maximized the following criterion:

$$score(binding\ site\ 1) + score(binding\ site\ 2) - 1.01 \times (spacing - 22), \tag{48}$$

where *score* of a binding sites is the number of base pairs matching the LuxO binding site (maximized over both LuxO binding sites), *spacing* is the distance between the binding sites in bp,

and where the 1.01 factor gives a slight advantage to optimal spacing when everything is equal. Using this analysis, we found 61 of 112 sequences with putative LuxO binding tandems and unique putative loop sequences, where we define loop sequences to be all bases between the last base of the putative tandem binding site and the first base of the putative promoter. This also allowed us to determine a putative consensus LuxO binding sequence to find bases at particular positions (depicted pictorially with the binding site logo graph in the inset of Fig. 6A): T T G C A A/T T/A T T G C A A.

7.3.3 AT/GC variation within the *qrr* enhancer sequences

The average relative identity values for both enhancer and upstream-enhancer sequences, averaged over all displacements (see horizontal lines in Figure 6C and 6D), are on the order of 0.25, which is the value expected for samples with equal probabilities for the four nucleotides at all loop positions, $P(\mathrm{A}) = P(\mathrm{C}) = P(\mathrm{G}) = P(\mathrm{T}) = 0.25$. Indeed, the mean value can be written more generally as: $\sum_x P(x)^2$, where x runs over all nucleotides A,C,G,T, which is greater than 0.25 for a non-uniform distribution. For the case of the *qrr* enhancer sequences, the distribution of nucleotides are $P(\mathrm{A,C,G,T}) = 0.3505, 0.1431, 0.1798, 0.3266$, indicating AT enrichment. In Figure 6C, we plot the AT and GC content as function of position within the loop sequences. The AT content is enriched at positions that are integer multiples of 10.6 bp, consistent with the helical periodicity of 10.45 bp found for the loop sequences using Fourier transform (FFT). While it is not clear what the significance of this enrichment is (e.g., sequences that are amenable for bending, binding site of some sort, etc.), it is interesting to note that we found no significant AT enrichment either at odd half-integer multiples of the helical periodicity, or for the non-looping enhancer upstream sequences.

7.4 Testing functionality of fusion proteins

To test additional protein size-related predictions of our model, we constructed a small set of fusion proteins, where a GST epitope was added at either the N-terminus or C-terminus of each of the TFs used in the experiment. All proteins were tested for functionality before a larger-scale synthetic enhancer experiment was performed.

7.4.1 TraR fusions

Fusion protein activity was tested by transforming cells with each of the pAct-Tra fusions as well as a synthetic enhancer bearing a TraR binding site. Both C-terminus and N-terminus fusions showed no effect upon 3OC8 induction. We concluded that the TraR-GST fusions lost their DNA binding ability and thus were not useful for our purposes.

7.4.2 LacI fusions

We chose to construct a C-terminus GST fusion, since it was reported that N-terminus fusions disrupt DNA binding of LacI and are inactive [29]. The C-terminus LacI-GST fusion were sequence verified, and its activity was tested in by transforming cells each of the pAct-LacI-GST fusions as well as a synthetic enhancer bearing a LacI binding site. In Supp. Fig. 8G, we compare the dose response functions for LacI-GST and LacI, for the LacI synthetic enhancer with the binding site set at $k = 95$ bp. Both dose response functions show a specific interaction with the LacI binding site, with the transition to unbound state occuring at nearly the same concentration of IPTG. The figure also shows that the expression level ratio is significantly smaller for LacI-GST as compared with LacI.

7.4.3 TetR fusions

His-TetR and His-GST-TetR activity were tested by transforming cells with each of the pAct-TetR fusions as well as a synthetic enhancer bearing a TetR binding site. DNA binding was further demonstrated in a gel shift assay (Supp. Fig. 6D).

Supplementary References

[1] O. Kratky, G. Porod. Röntgenuntersuchung gelöster fadenmoleküle. *Rec. Trav. Chim. Pays-Bas.* **68**, 1106–1123 (1949).

[2] Moroz, J. D. & Nelson, P. Torsional directed walks, entropic elasticity, and DNA twist stiffness. *Proceedings of the National Academy of Sciences* **94**, 14418–14422 (1997).

[3] Shimada, J. & Yamakawa, H. Ring-closure probabilities for twisted wormlike chains. application to DNA. *Macromolecules* **17**, 689–698 (1984).

[4] Marko, J. F. & Siggia, E. D. Stretching DNA. *Macromolecules* **28**, 8759–8770 (1995).

[5] Grassberger, P. Pruned-enriched Rosenbluth method: Simulations of θ polymers of chain length up to 1 000 000. *Physical Review E* **56**, 3682–3693 (1997).

[6] Pollak, Y., Goldberg, S. & Amit, R. Self-avoiding wormlike chain model for double-stranded-DNA loop formation. *Physical Review E* **90**, 052602 (2014).

[7] Chen, Z. Y. & Noolandi, J. Renormalization-group scaling theory for flexible and wormlike polymer chains. *The Journal of Chemical Physics* **96**, 1540–1548 (1992).

[8] Tree, D. R., Muralidhar, A., Doyle, P. S. & Dorfman, K. D. Is DNA a good model polymer? *Macromolecules* **46**, 8369–8382 (2013).

[9] Nepal, M., Yaniv, A., Shafran, E. & Krichevsky, O. Structure of DNA coils in dilute and semidilute solutions. *Physical Review Letters* **110**, 058102 (2013).

[10] Gennes, P.-G. *Scaling Concepts in Polymer Physics* (Cornell University Press, Ithaca, NY, 1979).

[11] Cloutier, T. E. & Widom, J. DNA twisting flexibility and the formation of sharply looped protein-DNA complexes. *Proceedings of the National Academy of Sciences* **102**, 3645–3650 (2005).

[12] Vafabakhsh, R. & Ha, T. Extreme bendability of DNA less than 100 base pairs long revealed by single-molecule cyclization. *Science* **337**, 1097–1101 (2012).

[13] De Pablo, J. J. & Escobedo, F. A. Monte Carlo methods for polymeric systems. In Prigogine, I. & Rice, S. A. (eds.) *Advances in Chemical Physics*, vol. 105, 337–367 (John Wiley & Sons, Inc., Hoboken, NJ, USA, 1998).

[14] Rappas, M., Bose, D. & Zhang, X. Bacterial enhancer-binding proteins: unlocking σ54-dependent gene transcription. *Current Opinion in Structural Biology* **17**, 110–116 (2007).

[15] Sanchez, A., Garcia, H. G., Jones, D., Phillips, R. & Kondev, J. Effect of Promoter Architecture on the Cell-to-Cell Variability in Gene Expression. *PLoS Comput Biol* **7**, e1001100 (2011).

[16] Sánchez, A. & Kondev, J. Transcriptional control of noise in gene expression. *Proceedings of the National Academy of Sciences* **105**, 5081–5086 (2008).

[17] Amit, R., Garcia, H. G., Phillips, R. & Fraser, S. E. Building enhancers from the ground up: A synthetic biology approach. *Cell* **146**, 105–118 (2011).

[18] Friedman, L. J. & Gelles, J. Mechanism of transcription initiation at an activator-dependent promoter defined by single-molecule observation. *Cell* **148**, 679–689 (2012).

[19] Ninfa, A. J., Reitzer, L. J. & Magasanik, B. Initiation of transcription at the bacterial glnAp2 promoter by purified E. coli components is facilitated by enhancers. *Cell* **50**, 1039–1046 (1987).

[20] Reitzer, L. J. *et al.* Mutations that create new promoters suppress the sigma 54 dependence of glnA transcription in Escherichia coli. *Journal of Bacteriology* **169**, 4279–4284 (1987).

[21] Sasse-Dwight, S. & Gralla, J. D. Probing the Escherichia coli glnALG upstream activation mechanism in vivo. *Proceedings of the National Academy of Sciences of the United States of America* **85**, 8934–8938 (1988).

[22] Rombel, I., North, A., Hwang, I., Wyman, C. & Kustu, S. The bacterial enhancer-binding protein NtrC as a molecular machine. *Cold Spring Harbor Symposia on Quantitative Biology* **63**, 157–166 (1998).

[23] Tu, K. C. & Bassler, B. L. Multiple small RNAs act additively to integrate sensory information and control quorum sensing in *Vibrio harveyi*. *Genes & Development* **21**, 221–233 (2007).

[24] Svenningsen, S. L., Waters, C. M. & Bassler, B. L. A negative feedback loop involving small RNAs accelerates *Vibrio cholerae*'s transition out of quorum-sensing mode. *Genes & Development* **22**, 226–238 (2008).

[25] Miyashiro, T., Wollenberg, M. S., Cao, X., Oehlert, D. & Ruby, E. G. A single qrr gene is necessary and sufficient for LuxO-mediated regulation in *Vibrio fischeri*. *Molecular Microbiology* **77**, 1556–1567 (2010).

[26] NCBI BLASTN. `http://blast.ncbi.nlm.nih.gov/blast/Blast.cgi?PROGRAM=blastn&PAGE_TYPE=BlastSearch&LINK_LOC=blasthome` (2015).

[27] Barrios, H., Valderrama, B. & Morett, E. Compilation and analysis of σ^{54}-dependent promoter sequences. *Nucleic Acids Research* **27**, 4305–4313 (1999).

[28] Lenz, D. H. *et al.* The small RNA chaperone hfq and multiple small RNAs control quorum sensing in *Vibrio harveyi* and *Vibrio cholerae*. *Cell* **118**, 69–82 (2004).

[29] Hammar, P. *et al.* The lac repressor displays facilitated diffusion in living cells. *Science* **336**, 1595–1598 (2012).
